# Artificial Tactile Perception System for Exploring Internal and External Features of Objects via Time‐Frequency Features

**DOI:** 10.1002/advs.202509928

**Published:** 2025-11-11

**Authors:** Yuanzhi Zhou, Jingqi Zhang, Linyi Li, Muxing Huang, Ziyi Zhang, Xingmin Lu, Zilan Li, Xuchun Gui, Xinming Li

**Affiliations:** ^1^ Guangdong Provincial Key Laboratory of Nanophotonic Functional Materials and Devices Guangdong Basic Research Center of Excellence for Structure and Fundamental Interactions of Matter School of Optoelectronic Science and Engineering South China Normal University Guangzhou 510006 China; ^2^ State Key Laboratory of Optoelectronic Materials and Technologies School of Electronics and Information Technology Sun Yat‐sen University Guangzhou 510275 China

**Keywords:** active exploration, artificial tactile perception system, object features cognition, time‐frequency features

## Abstract

Perceiving both external and internal object features is essential for accurate recognition and manipulation of humans and robots. However, relying on a single signal processing paradigm may hinder the extraction of specific tactile information from structurally similar signals, posing challenges in both accuracy and computational efficiency in artificial tactile perception systems. Active contact offers a means to modulate the mechanical interaction between tactile systems and objects, enabling targeted perception of specific properties. This work presents a flexible piezoelectric tactile sensing device with active perception capability. It exhibits high force sensitivity (<0.02 N), multi‐axis responsiveness, a wide frequency response range (upper than 2000 Hz), and high spectral resolution (<1 Hz). With two distinct active exploration motions (sliding and vibration), the system extracts edge features through time‐domain spike characteristics and infers internal contents via frequency‐domain decay trends. The device is integrated on the fingertip of a robotic dexterous hand, demonstrating its effectiveness in tasks such as texture recognition and liquid identification. It also shows promise for handling complex interaction tasks involving multiple subtasks. This study contributes to the advancement of tactile interaction paradigms in robotics and provides a foundation for more intuitive and adaptable robotic perception in human‐centered environments.

## Graphical abstract

By engaging in different active exploration behaviors, the tactile perception system can generate distinct signal modes, enabling the application of targeted decoding strategies for efficient and accurate perception of both external and internal object features. This approach facilitates a more advanced robotic understanding of human‐centered environments and supports learning‐based object recognition and manipulation.

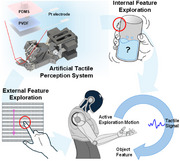

## 1 Introduction

Accurately perceiving the external and internal features of an object through touch is essential for understanding and manipulating the object,^[^ ^1^ ^]^ which enables robots to adapt to unstructured, human‐centric environments.^[^ ^2^, ^3^, ^4^, ^5^, ^6^ ^]^ Artificial perception systems have evolved to match human tactile cognition, enabling the ability to judge objects through the dynamic tactile signals generated during interaction, which will facilitate tactile perception and dexterous object manipulation. This demands broad frequency responsiveness and high sensitivity to minimize the blind spots of the tactile perception system.^[^ ^7^ ^]^ However, meeting these requirements in artificial tactile systems often results in increased structural complexity,^[^ ^8^, ^9^ ^]^ signal superposition,^[^ ^8^ ^]^ and unavoidable environmental noise,^[^ ^10^ ^]^ all of which reduce perceptual accuracy and impose greater computational burdens.^[^ ^11^, ^12^ ^]^

Current research mainly focuses on improving tactile perception systems’ mechanical sensitivity through structural optimization, such as bio‐inspired design^[^ ^13^, ^14^, ^15^, ^16^, ^17^, ^18^, ^19^ ^]^ or mechanical topology.^[^ ^20^, ^21^, ^22^, ^23^, ^24^, ^25^, ^26^, ^27^, ^28^, ^29^, ^30^ ^]^ Such approaches have proven effective in capturing external features such as object size,^[^ ^31^, ^32^ ^]^ contact force,^[^ ^6^, ^33^, ^34^ ^]^ and direction.^[^ ^31^, ^33^ ^]^ However, most existing designs are utilized in relatively low‐frequency scenarios and therefore struggle to capture internal object features, which are typically conveyed to the surface through high‐frequency mechanical waves. Although Meta reports a multimodal artificial tactile system, which could record signals over 3000 Hz, it shows capability in both external and internal feature perception.^[^ ^35^ ^]^ However, such a system incorporates multiple sensors based on different information modalities, which increases the complexity of subsequent data integration and processing.^[^ ^36^, ^37^ ^]^ In related fields, piezoelectric acoustic sensors have been successfully applied in speech recognition by detecting subtle vibrations of the neck skin during vocalization.^[^ ^38^, ^39^, ^40^, ^41^ ^]^ This demonstrates the advantages of high sensitivity and broad frequency responsiveness, making piezoelectric acoustic sensors possible to perceive both external and internal object properties using a single sensing unit, which could simplify system architecture and reduce the computation complexity. However, this introduces a new challenge in that both external and internal features are conveyed to a single sensing unit as dynamically varying force fields and are reflected as fluctuating amplitudes in responses. As a result, relying on a single signal processing paradigm may hinder the extraction of task‐specific tactile information from structurally similar signals, thereby compromising accuracy. As a reference, humans are capable of accurately perceiving object‐specific features by engaging in different forms of active exploration, following a mapping framework among exploratory behavior, processing strategy, and object characteristics.^[^ ^42^, ^43^, ^44^ ^]^ For a given exploratory behavior, the expected perceptual targets can be clearly defined, allowing neural systems to process tactile information according to predefined patterns.^[^ ^45^ ^]^ Therefore, by using active exploration motion to guide data processing, it is possible to achieve multimodal tactile perception based on a simplified artificial tactile sensing system.

Moreover, active exploration motion could modulate the mechanical interaction at the contact interface, shaping more distinguishable signal characteristics in both the time and frequency domains, thereby reducing the complexity of subsequent signal interpretation. In this work, we propose an active tactile perception method via time‐frequency features. Owing to the high sensitivity (<0.02 N), the wide frequency response range (upper than 2000 Hz), and the high spectral resolution (<1 Hz) of the proposed piezoelectric device, the artificial tactile perception system could extract external and internal features of objects by two distinct active exploration modes (sliding and vibration). It demonstrated the capability to distinguish edge distributions with a spatial resolution of less than 100 µm and an orientation resolution of less than 1°, showing its ability to capture external features. Additionally, the system responds to internal structural variations through distinct frequency‐domain signatures during contact. The system was shown to be capable of identifying objects with variations in both content type and volume. A series of real‐world scenarios, including grasping position detection, book flipping, and drink selection, demonstrate the system's potential to support robots in exploring and interacting with environments through active tactile perception, like how humans do. This design represents a step toward equipping robots with human‐like cognitive and manipulation capabilities, ultimately promoting their adaptation to human‐centered environments.

## 2 Active Tactile Perception and Device Design

**Figure 1:**
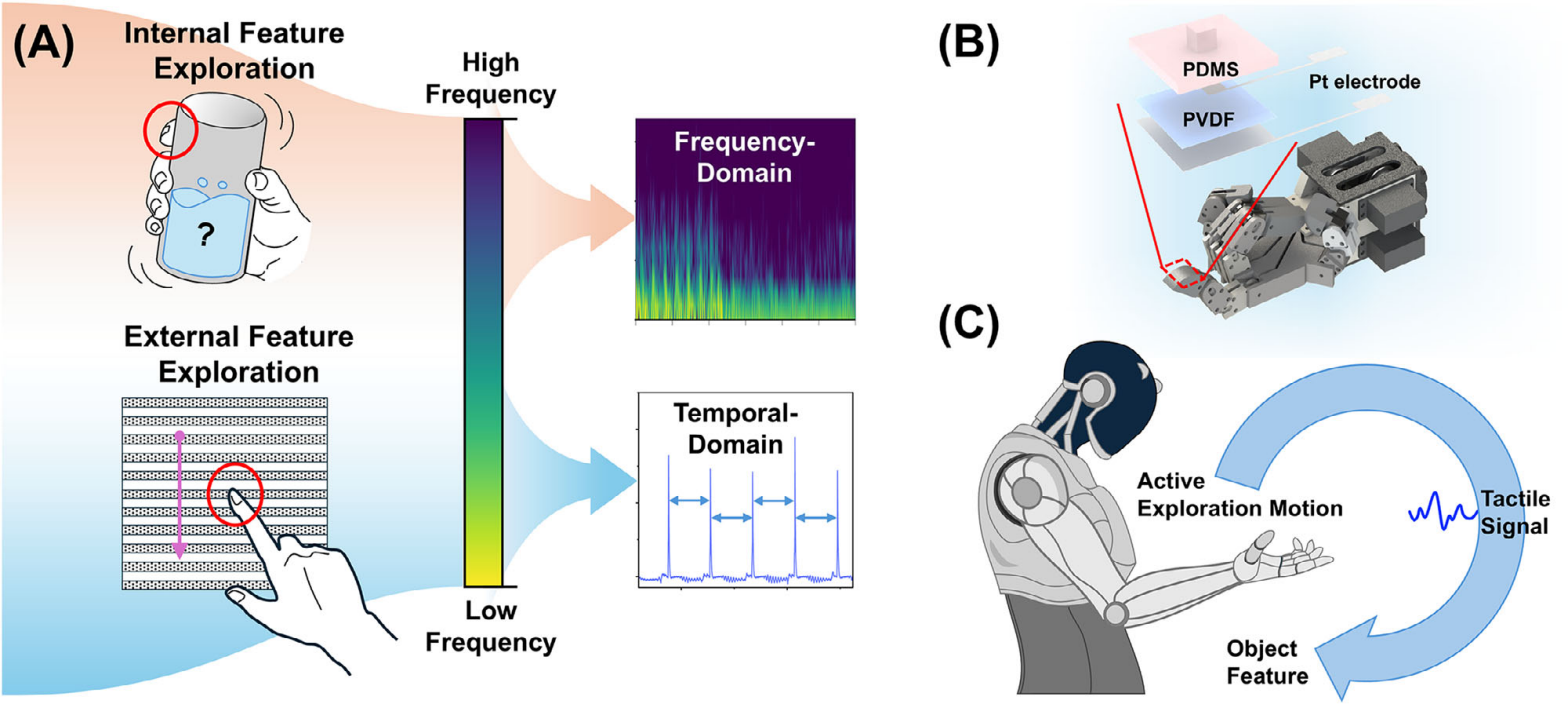
A) Active exploration motion: edge detection by sliding in the top left and content recognition by vibration in the bottom left; B) The diagram of the device structure; C) The artificial tactile perception system could help robots recognize object features by exploration.

Human tactile active exploration is characterized by the use of specific contact strategies to acquire information about the target object's properties,^[^ ^46^, ^47^ ^]^ mechanical interaction under different contact strategies could amplify the recognition ability of specific objects’ features. For example, fingers often perform slow tangential sliding along the surface of an object to perceive fine surface features through low‐frequency clues, while shaking behaviors are typically used to induce high‐frequency mechanical vibrations, which facilitate the inference of internal object properties^[^ ^48^, ^49^, ^50^ ^]^ (**Figure** **1**A). In this work, a PVDF (Polyvinylidene fluoride)‐based flexible piezoelectric sensing device with surface‐patterned pillar arrays was designed to realize the artificial tactile system that simulates human exploration of external and internal object features. The top layer of the sensing device is composed of PDMS (Polydimethylsiloxane)‐based pillars, which directly contact the object during active exploration. The pillars show different deformation modes according to specific active exploration motions, thereby inducing corresponding signal responses in the underlying PVDF layer (Figure 1B). The response signals, shaped by different exploratory behaviors, could be selectively decoded to achieve targeted perception of internal and external features (Figure 1C).

**Figure 2:**
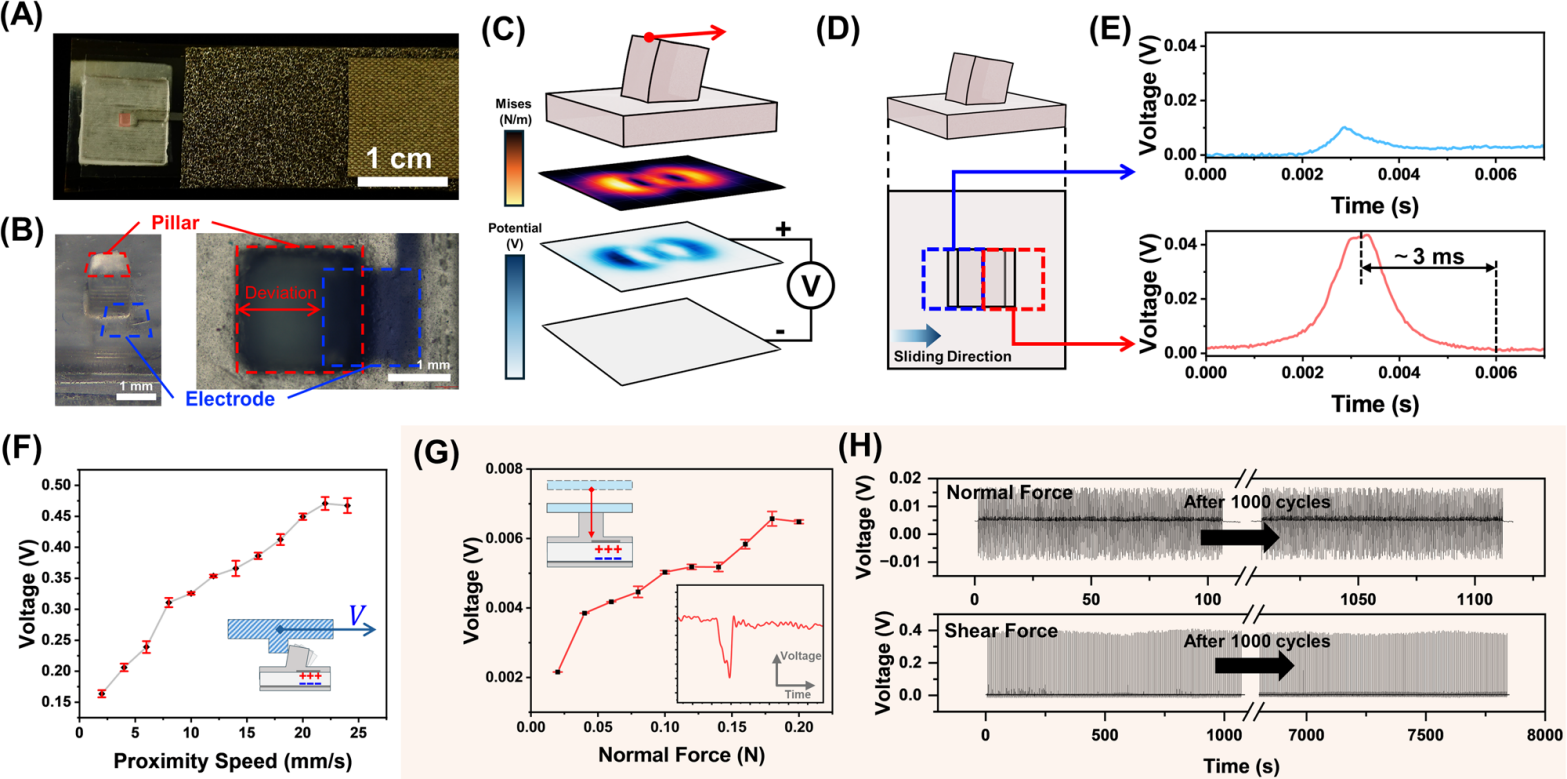
A) Photograph of the top view of the piezoelectric device, scale bar = 1 cm; B) Optical image showing the spatial alignment between the elastic pillar and the top electrode, scale bar = 1 mm; C) Simulated stress distribution in the PVDF layer when the pillar is subjected to tangential loading and corresponding electric potential distribution; D) two positions of the deviated‐located design: one is centered along the axis of compression (red dashed box), and the other is centered along the axis of stretching (blue dashed box); E) Comparison of the resulting signals within the PVDF layer at the two positions; F) The response to tangential sliding with different velocity (from 2  to 20 mm s^−1^); G) The response to normal force (from 0.02 to 0.2 N); H) The repeatability of the device during 1000 cycles.

**Figure** **2**A shows the top view of the sensing device of the artificial tactile system. The elastic pillar is highlighted with red rectangle in the figure. A PVDF layer and electrode layers were placed beneath the elastic pillar layer. To evaluate the intrinsic piezoelectric response of the PVDF layer, impact tests were conducted using weights of different masses dropped from the same height, as shown in Figure S1 (Supporting Information). Considering sliding as a typical exploration scenario, a deviated‐located design of the top electrode was introduced based on the charge distribution on the PVDF film during sliding. Specifically, each electrode in the top electrode layer adopts a deviated‐location design relative to its corresponding pillar (Figure 2B), enabling a directional response along the sliding direction. In contrast, the bottom electrode layer is uniformly coated with platinum, serving as a common ground. The fabrication process of the sensing device is illustrated in Figure S2 (Supporting Information). The SEM image of the pillar and the electrode could be found in Figure S3 (Supporting Information).

Based on the deviated‐located design, the deviated electrode could align with the region of maximal charge variation during directional sliding. This enables direction‐specific responses in the form of pronounced spike signals. As shown in Figure 2C, when sliding occurs, the strain is predominantly concentrated around the two lateral edges of the pillar that are perpendicular to the sliding direction. At this time, one side is subjected to tensile stress, and the other side is subjected to compressive stress. This asymmetric stress distribution causes the in‐plane electric displacement within the PVDF layer. The electric potential on the compressed side is positive, and the one on the stretched side is negative, with the bottom surface of the PVDF defined as the zero‐potential reference. As a result, the electric charge redistributes within the PVDF layer around the lateral edges of the pillar. Figure 2D shows two positions of the deviated‐located design: one is centered along the axis of compression, and the other is centered along the axis of stretching. The top electrode pattern to be centered along the axis of compression could enable specific piezoelectric response along the sliding direction (≈ 3 times to the opposite side), as demonstrated in Figure 2E. Besides, the signals from the centered‐located design of the top electrode and pillars are illustrated in Figure S4 (Supporting Information). The piezoelectric response maintains for ≈ 3 ms from generation to disappear, which shows the potential to record high‐frequency tactile information.

Considering different exploration methods would influence the kind of contact of the pillar, the response of the device to the different contacts was characterized. Figure 2F demonstrates the response of tangential sliding with different velocities. With increasing proximity velocity, the response amplitude of the device gradually increases and tends to saturate at ≈ 470 mV. This is presumed to result from higher velocities causing larger instantaneous stress variations, further generating more pronounced electric displacement. Figure 2G shows the device's response to the normal force, exhibiting a clear linear increase under stepwise loading with 0.02 N increments, demonstrating its fine mechanical responsiveness. For complex interactions involving coupled normal and tangential forces, the device output is jointly modulated by contact depth and sliding speed, as shown in Figure S5 (Supporting Information). Figure 2H demonstrates the long‐term stability of the device, as confirmed by 1000 cyclic loading tests conducted under both tangential and normal force conditions. Table S1 (Supporting Information) provides a quantitative comparison of key performance metrics between this work and related studies. From this comparison, it could be inferred that the proposed device demonstrates both comprehensive performance and high sensitivity.

However, to achieve the tactile perception of the external and internal features of the target objects, the sensing signal requires not only a recognizable amplitude but also well‐defined waveform characteristics to establish a clear mapping to object features. The waveform characteristics could be modulated by designing the structure of the pillar. The geometric structure plays a critical role in determining the pillars’ mechanical properties, including stiffness, natural frequency, and damping ratio, thereby shaping their deformation response to excitations during active exploration, which is ultimately reflected in the time‐frequency features of the signals from the PVDF layer. Based on the previously established electrode–pillar alignment, the influence of pillar geometry on signal modulation was further investigated by comparing a series of square pillars with width‐to‐height ratios of 1:0.5, 1:1, 1:1.5, and 1:2 mm under tangential mechanical stimulation. To ensure consistent excitation conditions across different structures, the interaction depth between the contacting object and the pillar was controlled to be half the pillar height, thereby maintaining uniformity in relative contact position and excitation energy.

**Figure 3:**
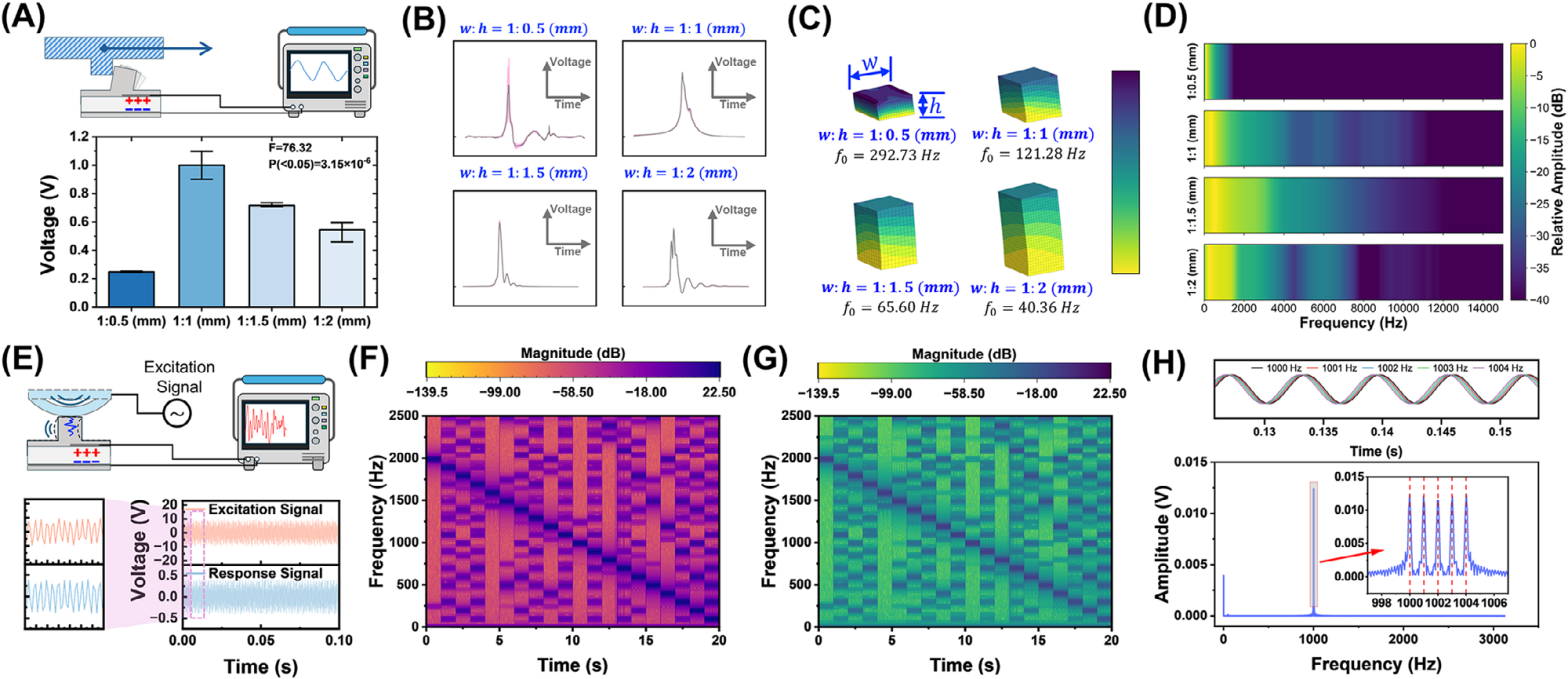
A) Schematic of the sliding test and comparison of the signal amplitudes modulated by elastic pillars with 4 structural ratios (width: height = 1:0.5 to 1:2 mm). B) Comparison of the corresponding normalized waveforms; C) Simulated natural frequencies of elastic pillars with 4 structural ratios under fixed boundary conditions; D) Corresponding frequency‐domain representations of the signals; E) The schematic diagram of the vibration test; F) The STFT (Short‐Time Fourier Transform) spectrogram of the excitation signal; G) The STFT spectrogram of the response signal; H) Time‐domain diagram of multi‐frequency excitation (top) and FFT (Fast Fourier Transform) spectrum of the corresponding response signal (bottom).

As shown in **Figure** **3**A, the response amplitude of the structure with an aspect ratio of 1:0.5 mm is significantly attenuated compared to structures with higher aspect ratios, making it more susceptible to noise‐induced errors. To ensure that this observation is not due to random fluctuations, we evaluated repeatability across multiple devices. The error bars represent the variability among samples, with the maximum signal difference not exceeding 0.1 V, indicating good consistency. Statistical analysis (F = 76.32, P = 3.15 × 10^−6^ < 0.05) further confirms that the variations are highly significant and primarily associated with structural design rather than noise, supporting the robustness of the device performance.

The normalized waveforms in Figure 3B further confirm this trend. Moreover, taller pillars tend to introduce longer oscillation periods in the modulated piezoelectric signals, increasing the possibility of waveform overlap or signal miscounting.^[^ ^51^ ^]^ As illustrated in Figure 3C, for a fixed pillar width, increasing the height reduces the natural frequency from 292.73  to 40.36 Hz. This trend aligns with the results in Figure 3B because lower natural frequencies usually correspond to greater susceptibility to contact‐induced resonance, leading to oscillation in signal responses. The frequency‐domain analysis in Figure 3D demonstrates that the signals modulated by pillars with width‐to‐height ratios of 1:0.5 and 1:1 mm are primarily concentrated in the low‐frequency range, whereas higher‐ratio structures distribute energy over a broader frequency spectrum. We believe that the low‐frequency characteristics of the signal could be associated with the mechanical deformation occurring with the spike timing mechanism during the sliding process.^[^ ^52^ ^]^ As this relationship is less affected by artifacts from resonance‐induced interference, the features could be extracted using simple threshold‐based algorithms without the need for complex feature processing techniques. Based on this balance of amplitude stability and frequency characteristics, the 1:1 mm pillar configuration was selected for subsequent experiments.

Since active exploration motions generate signals across a wide frequency range, it is critical to evaluate the frequency response of the sensing device. Thus, a piezoelectric actuator was mounted in fine contact with the pillar. When excited by a periodic signal, the actuator's vibration would be transmitted through the pillar to the underlying PVDF layer, resulting in a corresponding electrical output, as shown in Figure 3E. Figure 3F,G shows the excitation signal and the corresponding response signal in the frequency domain, respectively. The results indicate that the sensing device effectively captures and transmits subtle mechanical vibrations within the frequency range of 100  to 2000 Hz, exhibiting highly consistent features in the frequency spectrum. Figures S6 and S7 (Supporting Information) compare the time and frequency domain signal responses of different structures under high‐frequency stimulation, respectively. To further validate this spectral consistency, a composite excitation consisting of multiple superimposed sine waves was applied to investigate the device's response to complex frequency components. The result shows that even if the superimposed high‐frequency inputs were close in the frequency domain (with differences as small as 1 Hz), the response signal from the device exhibited clear spectral components corresponding precisely to each input frequency, as shown in Figure 3H. This indicates that the structure is capable of accurately capturing high‐frequency information, suggesting its potential as an artificial tactile perception system for inferring internal object properties through active exploration.

## 3 Characterization & Analysis in Active Tactile Perception Ability

### 3.1 External Information Perception: Edge Detection

**Figure 4:**
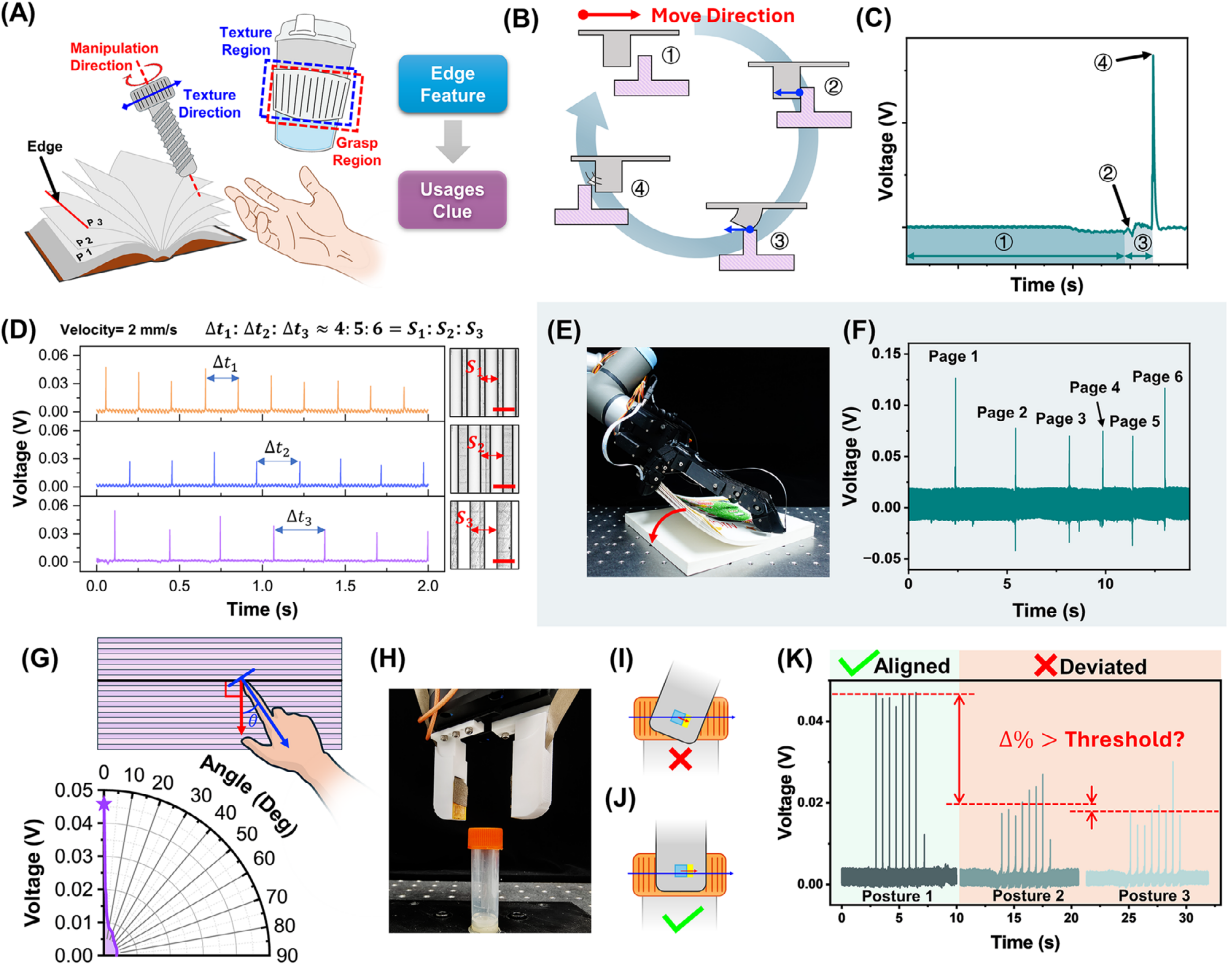
A) The importance of edge detection: Functional external features guide user interaction through structured edge distribution; B) Schematic of the elastic pillar's interaction with single edge during sliding and C) the corresponding signal; D) Comparison of signal in temporal domain with different edge intervals (400 , 500 , and 600 µm), scale bar = 500 µm; E) Photograph of a robotic hand to turn pages of a book with the device integrated on the thumb; F) The distinct signal spikes precisely corresponding in both timing and count to each page being turned; G) Response to different angle between slide direction and edge direction in 0–90 degree; H) Photograph of a two‐finger robotic hand to unscrew the cap of a biological sample tube, with the devices on the fingertip; I,J) The schematics about the relationships among rotation axis, the central axis of the cap, and the edge distribution direction on the side wall of the cap; K) Comparison of device responses under axial alignment and deviation.

Recognizing the periodicity and orientation of edge distributions of an object could help tactile perception infer object functionality and guide interactive behavior. As illustrated in **Figure** **4**A, edges’ distribution on objects’ surfaces is a key external feature due to its close association with objects’ intended functions and modes of operation.^[^ ^53^, ^54^, ^55^ ^]^ For example, the edge of a page is related to the book's page number. Besides, the periodic distribution of multiple edge structures along a principal direction forms textures. The texture on the screw usually indicates its rotation axis; and the texture region on containers guides for humans to grasp without falling. Thus, through sliding excitation, we investigated the artificial tactile device's capability for edge detection and demonstrated its potential for exploring external object features to guide manipulation in realistic scenarios.

A simplified test scenario was established to investigate the edge detection capability of the device: the number of elastic pillars on the device surface was set to one, and the device was fixed. A single edge‐shaped protrusion was moved at a constant speed of 2 mm s^−1^ using a motorized stage to apply a tangential force to the elastic pillar. The piezoelectric signal was continuously recorded throughout the entire process, from the moment the edge began approaching the pillar until it completely separated from the pillar. Figure 4B illustrates the contact process of an elastic pillar interacting with each edge structure through tangential sliding. The corresponding signal output is shown in Figure 4C. First, before the contact occurs, the signal remains stable. Then, as the elastic pillar encounters the edge, it undergoes gradual deformation due to the combined effects of bending and shear forces. Because of the elasticity of the pillar, the deformation process is maintained until the whole pillar comes to the top surface of the edge, resulting in a small signal fluctuation. As the pillar slides over the edge top surface, it maintains a relatively constant deformation, which is reflected in a stable signal output. Finally, at the moment the pillar disengages from the edge, a rapid release of stored elastic energy induces a sudden displacement field in the PVDF layer, generating a distinct signal spike. This spike corresponds to the moment the pillar loses contact with the edge.

In real‐world scenarios, the signal characteristics during this process, shown in Figure 4C, may vary quantitatively depending on the contact conditions. For example, different sidewall angles could amplify the fluctuations observed in Stage 2 that are induced by contact. This highlights the need for subsequent signal processing methods to be adapted to real‐world phenomena to minimize potential misinterpretations. In addition, differences in the protruding edge materials may also affect the morphology of the Stage 4 spike due to variations in elastic modulus. To evaluate this effect, we tested the device response using 6 types of contact probes under the same normal displacement and tangential sliding speed (Figure S8, Supporting Information). For materials with an elastic modulus not lower than that of the device itself (Resin, PMMA, Wax, and PDMS), the spike signals and amplitudes showed no significant differences. In contrast, for softer materials with lower elastic modulus (Dragon Skin 30 and Ecoflex 30), the viscoelastic behavior often introduced additional energy dissipation during the mechanical contact process, resulting in reduced peak amplitudes and even sustained oscillations even after the probe detached from the device.

To further confirm the specificity of this response to the edge (rather than roughness), the device was slid on a series of flat substrates without lubrication, including a mirror‐finished glass slide, a polished silicon wafer, and an unpolished silicon wafer (Figure S9, Supporting Information), as well as on probes covered with sandpapers of graded roughness ranging from 400 to 2000 grit (Figure S10, Supporting Information). These surfaces are supposed to provide a representative span of roughness levels, covering representative roughness gradients. The results indicate that although surface roughness could introduce minor fluctuations, the overall signal amplitude remains nearly unaffected. Specifically during continuous sliding across a series of sandpapers in Figure S10 (Supporting Information), the signal amplitude induced by surface roughness is nearly indistinguishable from the noise observed in the resting state (blue region) and remains far smaller than the pronounced spike signals generated when leaving the sandpaper edge. This result supports the perspective that the device is selectively responsive to edge features, rather than general surface contact or friction.

As discussed earlier, the pillar with a width‐to‐height ratio of 1:1 mm enables the device's signal to concentrate in the low‐frequency range, thereby allowing signal spikes to correspond one‐to‐one with individual edges on the objects’ surface. By analyzing the spacing and density of these signal spikes, the periodic distribution of edges on objects’ surfaces can be inferred. As shown in Figure 4D, when sliding on the surface structures with protrusion spacings of 400, 500, and 600 µm, the device accurately estimated the spacing based on the temporal intervals between signal spikes, with a normalized error of less than 2.62% (Figure S11, Supporting Information). In addition, since edges typically occur at the transition between raised and recessed regions on a surface, the device's edge detection capability was further investigated by varying the widths of these regions, as shown in Figures S12 and S13 (Supporting Information). The results showed that when the raised region was as narrow as 100 µm, the device could still reliably detect edge textures, with clearly identifiable signal spikes. However, when the recessed region was narrowed to 100 µm, the device no longer produced recognizable spikes. This may be due to the limited rebound of the elastic pillar at the moment of release, as the narrow recess restricted deformation recovery, resulting in weak or unobservable piezoelectric signals. Overall, these findings indicate that the device can effectively detect the edge of a raised structure even when it is very narrow, but excessively narrow recessed regions may suppress signal generation.

This response capability to narrow protrusions provides potential for precise edge detection tasks. As shown in Figure 4E, the device was integrated into the thumb of a robotic hand as an artificial tactile perception system, enabling recognition of individual pages during slight book‐turning motions. (Video S1, Supporting information). As thin and lightweight pages gently slide across the pillars, distinct signal spikes are generated, precisely corresponding in both timing and count to each page being turned (Figure 4F), which indicates that the artificial tactile perception system could be used to guide robots in gently interacting with delicate objects, such as paper, cash, or clothing, by external edge features.

In addition, the proposed device design also exhibits a distinct directional sensitivity to edge orientation. As shown in Figure 4G, a misalignment as small as 1° leads to a reduction in signal peak amplitude by more than 50%. This phenomenon can be attributed to two main factors. First, there may be a spatial offset between the center of the elastic pillar and the center of the electrode, resulting in reduced signal coupling at off‐angle orientations. Second, as the contact angle increases, the pillar can no longer disengage cleanly from the edge protrusion. Instead, it undergoes a gradual release accompanied by torsional deformation, which reduces the rate of change in the electric displacement field and thereby weakens the signal peak. This feature is important to those manipulations sensitive to angles. We demonstrate a scenario that uses a two‐finger robotic hand to unscrew the cap of a biological sample tube, as shown in Figure 4H. The key to successfully unscrewing the cap is to ensure that the rotation axis of the robotic hand is critically aligned with the cap's center axis, which could be inferred by the textures on the cap because the edge distribution direction is usually vertical to the center axis (Figure 4I,J). By sliding along the cap sidewall, the robotic hand could utilize the signal output amplitude as a clue to align with the cap— only if the robotic hand's rotation axis is exactly aligned with the cap's central axis, the artificial tactile perception system produced a markedly higher amplitude, as shown in Figure 4K. Thus, by comparing the relative difference between adjacent sliding angles, a threshold could be defined to infer the exact rotation axis. This threshold‐based approach yielded an orientation discrimination accuracy of 89.5% in our experiments.

In summary, this section discussed the capability of the proposed artificial tactile perception system to perceive edge distribution features on object surfaces through spike‐based signal features. This capability stems from a robust mapping between the generated spike signals and the edge release events. Such perception is expected to assist robots in rapidly forming an understanding of object properties and functions.

### 3.2 Internal Information Perception: Content Recognition

**Figure 5:**
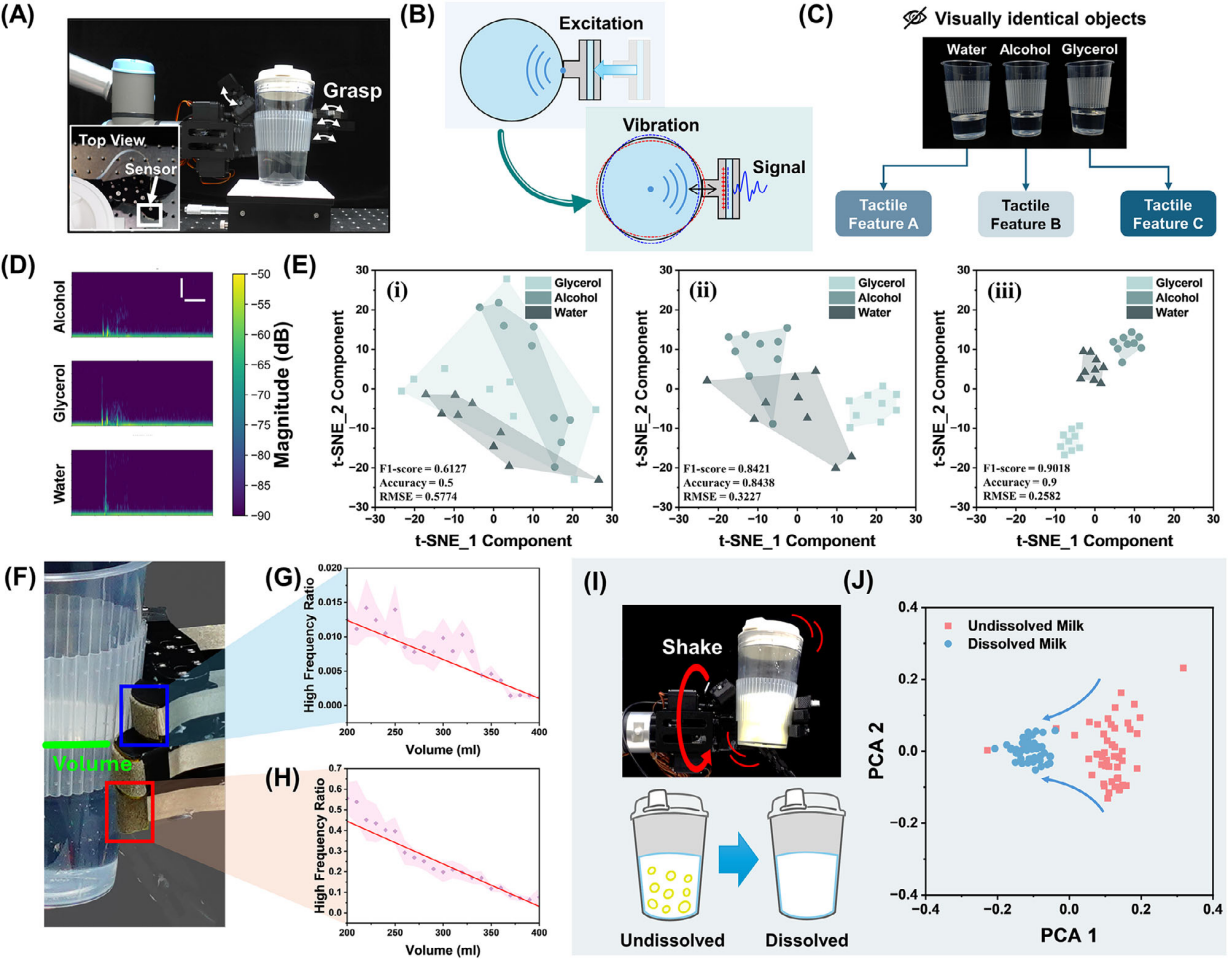
A) Device integrated into a robotic fingertip to simulate human‐like tapping or shaking by grasping; B) The schematic diagram of mechanical excitation applied to the coupled vibration system; C) High‐frequency signal response of the PVDF layer triggered by the coupled vibration system at the moment after excitation; D) Time–frequency signal characteristics of cups filled with water, alcohol, and glycerol under grasping excitation, vertical scale bar = 500 Hz, horizontal scale bar = 0.5 s; E) t‐SNE‐based 2D visualization of frequency‐domain features for water, alcohol, and glycerol under increasing excitation levels (i–iii): grasping only, shaking ×3, and shaking ×6; F) The photograph of the robotic hands grasp a container filled with specified volume water; G,H) The high frequency ratios change with various volume of water without/with cup sleeves; I) The photograph and the schematic diagram of shaking the cup to dissolve the milk powder; J) PCA‐based 2D visualization of feature distributions for dissolved and undissolved milk samples.

To emulate the strategies of human hands to explore internal object properties, such as tapping or shaking, the device was integrated into the fingertips of a robotic hand (**Figure** **5**A). During the grasping process, the elastic pillars of the device came into full contact with the cavity surface. As shown in Figure 5B, this interaction served as a mechanical excitation to the coupled vibrating system formed by the cup and its contents, inducing vibrations of the coupled vibrating system. These vibrations would gradually damp out after the external excitation ceased for a while. Throughout this process, the elastic pillar functioned as a transmission medium for mechanical vibration waves, simultaneously modulating the stress applied to the PVDF layer and inducing high‐frequency signal responses. As the vibrations decay, the frequency‐domain signal attenuates over time, and the rate of this decay is closely linked to the properties of the contents. In the case of liquids, viscosity determines energy dissipation, surface tension affects wave propagation across the liquid surface, and liquid height significantly influences the mass distribution and modal frequency structure.^[^ ^56^ ^]^ Thus, the frequency‐decay trend could be considered as a kind of tactile feature to extract objects’ internal features (Figure 5C).

Different liquid types’ influence on the artificial tactile perception system's signal frequency feature was investigated under identical volume and container conditions. Alcohol, glycerol, and water were selected as 3 typical liquid types due to their significant difference in viscosity and surface tension. The robotic hand grasped the cup and remained still until the vibrations were no longer observable to the naked eye. The signals obtained during this period were analyzed using a short‐time Fourier transform (STFT) and were plotted as time‐frequency maps. For a complete analysis of the signal in the frequency domain, the sampling rate was set to 50000 Hz to capture signal features over a broad frequency range. As shown in Figure 5D, the frequency‐domain responses of alcohol, glycerol, and water were primarily concentrated below 1500 Hz, with a significant portion located in the low‐frequency range under 500 Hz. Among them, the signals from alcohol and glycerol exhibited prolonged vibration durations and more complex frequency‐domain patterns that lacked consistent temporal trends, suggesting more intricate vibrational behavior compared to water. Due to its high viscosity (viscosity_glycerol_ = 934 mPa·s while viscosity_alcohol_ = 1.074 mPa·s and viscosity_water_ = 0.89 mPa·s at 25 °C^[^ ^57^ ^]^), glycerol dissipated kinetic energy more slowly, thereby sustaining a broader range of frequency components over a longer period. In contrast, water's frequency oscillations decay more quickly, attributed to its lower viscosity and faster energy dissipation. Although alcohol and water have similar viscosities, alcohol's lower surface tension (surface tension_alcohol_ = 21.82 mN m^−1^ while surface tension_water_ = 72 mN m^−1^ and surface tension_glycerol_ = 62.5 mN m^−1[^ ^57^ ^]^) made it harder to return to a stable state after excitation, resulting in a more chaotic and persistent broadband response.

Accurate classification of different liquids requires the artificial tactile perception system to amplify subtle frequency variations induced by vibration. Although alcohol and glycerol differ in viscosity and surface tension, their time‐frequency responses under grasp‐induced excitation exhibit similar distributions. Therefore, liquid content recognition based on grasp‐induced excitation is limited, indicating that this mode is not robust enough to generate distinguishable features for the artificial tactile perception system. To address this limitation, shaking behavior is introduced as an enhanced form of excitation based on grasping. Figure 5E illustrates the distribution of features extracted from the piezoelectric signals of alcohol, glycerol, and water under different excitation behaviors. Figures S14 and S15 (Supporting Information) present the artificial tactile perception system's time‐domain signals and the corresponding frequency‐domain responses under different stimulation behaviors. As shown in Figure 5E (i), the feature region of glycerol in the 2D space largely overlaps with those of both water and alcohol under grasping‐induced excitation. In contrast, the feature regions of water and alcohol show less overlap with each other. This observation is consistent with the previous analysis that, although grasping can provide a basic differentiation among liquid types, it may lead to misclassification.

To enhance the excitation, the container was subsequently shaken 3 times at a fixed amplitude and frequency after grasping. The resulting feature distribution, shown in Figure 5E (ii), reveals a noticeable contraction in the glycerol feature region, indicating that the introduction of shaking made the frequency features of glycerol more distinguishable from the other two liquids. This may be attributed to the low‐frequency, periodic shaking causing slower kinetic energy dissipation in glycerol due to its high viscosity, thereby enhancing its distinctiveness in the frequency domain. This finding is further supported by the Short‐Time Fourier Transform (STFT) results in Figure S6 (Supporting Information). When shaking cycles are further increased to 6, all three liquids exhibit clustered feature distributions in the 2D space, as shown in Figure 5E (iii), reflecting well‐separated frequency‐domain characteristics. Based on the K‐means clustering results, introducing three shaking cycles improved the recognition accuracy for the three liquids from 0.5 to 0.8438, accompanied by an increase in the F1 score from 0.6127 to 0.8421 and a reduction in Root Mean Square Error (RMSE) from 0.5774 to 0.3227. When the shaking cycles were further increased to 6, the accuracy rose to 0.9, with the F1 score reaching 0.9018 and the RMSE decreasing to 0.2582. These results demonstrate that, with appropriate excitation strategies, the artificial tactile perception system can effectively distinguish different liquid contents inside the container.

In addition, differences in liquid volume within the container can also affect the signal collected by the artificial tactile perception system. The artificial tactile perception system responses under grasping‐induced excitation with liquid volumes ranging from 200  to 400 mL were recorded (Figure 5F). As previously described, the frequency‐domain responses were primarily concentrated below 1500 Hz, particularly in the low‐frequency region under 500 Hz. At the moment of grasping, high‐frequency components exceeding 500 Hz were observed, while interference signals caused by mechanical vibrations of the robotic hand were mainly distributed above 1500 Hz (Figure S16, Supporting Information). Therefore, 0–1500 Hz was defined as the analysis range, and the proportion of high‐frequency energy ratio within the 500–1500 Hz band was calculated. As shown in Figure 5G,H, the proportion of high‐frequency components in the signal decreases progressively with increasing liquid volume, both with and without the presence of a cup sleeve. This trend is likely due to the increased liquid mass and internal viscous dissipation, which dampen high‐frequency vibrations induced by grasping. The cup sleeve further attenuates mechanical vibrations, as reflected in the overall lower high‐frequency content in Figure 5G compared to Figure 5H. Nonetheless, the consistent downward trend across both conditions indicates that the volume‐related attenuation trend of the high‐frequency energy ratio is robust to external damping effects.

Given that grasping and shaking a container is common in tactile interactions, the artificial tactile perception system could further evaluate the internal characteristics of the container. For instance, milk powder usually requires a long period of shaking to completely dissolve in water. However, it is difficult to visually distinguish between undissolved powder clumps and fully mixed milk (Figure 5I). During the shaking process, differences in dissolution states may lead to variations in the liquid's center of mass, surface tension, and viscosity. These factors may cause the coupled vibration system to respond differently to identical mechanical stimuli. Based on the sensor's design, it is possible to detect such differences in mechanical response by analyzing the frequency‐domain characteristics of the piezoelectric signals generated during shaking (Video S2, Supporting information). To validate this, frequency domain features were extracted for a series of time periods, and the resulting feature distributions were visualized in a 2D space, as shown in Figure 5J. The undissolved samples are more scattered in space, reflecting the evolving physical properties of the solution during each shake. The dissolved samples form a compact cluster in the feature space, indicating consistent signal characteristics when the milk is fully dissolved and behaves as a uniform medium.

In summary, this section demonstrates that the proposed artificial tactile perception system, with its broad frequency response and high spectral resolution, could detect subtle vibrations induced by grasp or shake, enabling the classification of both the type and volume of internal contents. It could be inferred that, with more advanced feature extraction techniques, the proposed artificial tactile perception system could be extended to recognize a broader range of object types and internal properties.

## 4 External and Internal Information Dual‐Mode Perception

**Figure 6:**
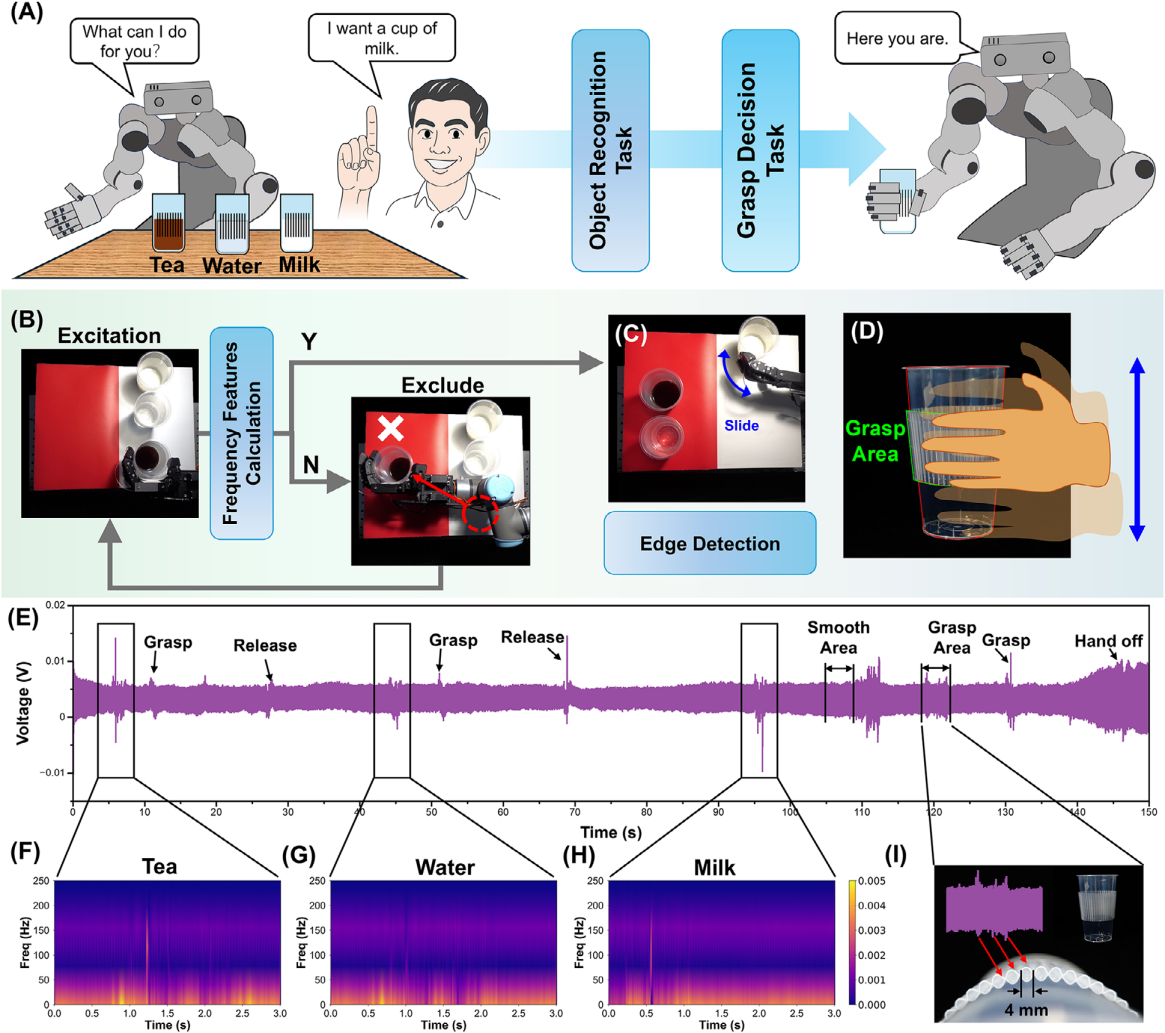
A) Scenario setup: responds to a customer's request by selecting the desired beverage from a variety of drinks on the table and delivering it to the customer, including two subtasks; B,C) The entire workflow of the robotic bartender performing the task; D) The schematic diagram of grasp decision; E) The complete artificial tactile perception system signal recording throughout the task; F–H) Representative responses in the frequency domain for three different beverages (tea, water, and milk); I) Response to texture clue from grasp area.

Based on the above results, the proposed artificial tactile perception system has been shown to capture both time‐domain and frequency‐domain features via various active exploration motions as excitations, enabling the perception of both external and internal object properties. With the help of the artificial tactile perception system, the robot can actively select appropriate excitation strategies based on task context and environmental requirements, thereby enabling adaptive characterization of object features. This facilitates informed decision‐making for subsequent actions, similar to how humans perform complex tasks through a sequence of interrelated subtasks. In this section, a conceptual application scenario is presented to demonstrate how the artificial tactile perception system enables robots to perform complex interactive tasks. **Figure** **6**A illustrates how a robotic bartender responds to a customer's request by selecting the desired beverage from a variety of drinks on the table and delivering it to the customer. This task involves two subtasks, each posing specific cognitive demands on the robot. Specifically, the first subtask is an object recognition task, which requires the robot to accurately identify the type of liquid in each cup and choose the right one according to the customer's request. The second subtask is a grasping decision task, in which the robot must select an appropriate grasping strategy based on the cup's characteristics to ensure secure delivery.

Figure 6B,C illustrates the entire workflow of the robotic bartender performing this task. The corresponding presentation can be found in Video S3 (Supporting Information). For safety considerations, the robot uses grasping rather than shaking to excite the coupled vibration system, and the returned frequency information is then used to make a rough estimation of the liquid type. In the demonstration, the robot moves mismatched drinks to a red‐labeled area to provide intuitive visual feedback, signaling that they do not match the user's request. Once the target drink is identified, the robot proceeds to determine an appropriate grasping strategy based on edge detection along the cup's sidewall. Specifically, the graspable area of the cup is typically designed with anti‐slip textures. When the fingertip slides across these textured surfaces, a sequence of distinct signal spikes of the artificial tactile perception system is generated due to the distribution of texture edges, allowing the robot to identify a suitable gripping region (Figure 6D).

By using the proposed artificial tactile perception system to record the complete signal during the entire task, the object‐related information and the interaction process with the robot are reflected, as shown in Figure 6E. During complex interaction tasks, occasional minor collisions are practically unavoidable, which may introduce additional noise into the signal. These noises are not separately annotated in the figure. Although their morphology may resemble valid responses, they are not included in subsequent processing because our analysis is restricted to signals occurring within defined windows of given exploratory behaviors. The signals generated during grasping excitation reveal distinct differences in the frequency‐domain responses of various beverages, providing informative clues for the robot to identify and select the drink requested by the customer (Figure 6F–H). Figure 6I further illustrates the relationship between the sharp signal spikes and the textured grasp regions on the cup surface. In contrast, the smooth, non‐graspable areas do not elicit such spike features in the sensor signal, indicating their unsuitability for secure grasping. Based on the proposed artificial tactile perception system, robots could identify safe grasping positions by analyzing the characteristics of spike signals.

## 5 Conclusion

This work proposes a flexible PVDF‐based piezoelectric tactile sensing device with a surface pillar layer. By employing two distinct active exploration motions to induce corresponding deformation modes, the system enables frequency‐domain separation of external and internal object features, thereby achieving dual‐mode perception. It could use the time‐domain spike series caused by sliding as an external object feature clue for edge detection, and the vibration‐induced frequency‐decay trend as an internal object feature clue for content characterization. By encoding diverse tactile information into a multidimensional data space, the artificial tactile perception system enables robots to effectively handle complex task flows in realistic and well‐defined human‐robot interaction scenarios. Importantly, this capability does not rely on algorithmic decoupling of sensory information. Instead, it is grounded in a human‐like perception mechanism that uses active exploration motions and tactile feedback jointly as inputs that guide signal processing in a context‐appropriate manner. Looking ahead, this mechanism could be further extended by incorporating multimodal sensing modalities to enhance the perceptual capacity of artificial tactile systems. Building on the present signal feature analysis, future work may combine this framework with advanced algorithms to facilitate the real‐time execution of complex tasks in real‐world, unstructured environments.

## 6 Experimental Section

### The Device Fabrication

First, the PDMS pillar layer was prepared using a custom‐designed 3D‐printed mold. The PDMS precursor (Dow Corning Sylgard 184) was mixed at a 10:1 ratio (base: curing agent), thoroughly stirred, and cast into the mold. The mixture was then cured in an oven at 70 °C for ≈ 6 h before demolding. A commercial PVDF film with a thickness of 28 µm (TE Connectivity) was used as the sensing layer. Platinum electrodes were deposited on both sides of the PVDF film using magnetron sputtering (Quorum Q150TSplus). The PDMS pillar layer was bonded to the PVDF film using a thin spin‐coated PDMS adhesive layer and cured. After curing, the structure was encapsulated with a polyethylene terephthalate (PET) film for mechanical protection. To reduce electromagnetic interference, conductive shielding was applied using metallic adhesive tape.

### Electrical Measurement

All electrical measurements were conducted using an MSO44 series oscilloscope (Tektronix). For basic performance characterization of the sensor, conductive wires were used to directly connect the sensor electrodes to the oscilloscope for signal acquisition. In subsequent experiments involving integration with a robotic manipulator, the electrodes were routed through an interface compatible with flexible printed circuit (FPC) connectors. A custom‐designed printed circuit board (PCB) and coaxial cables were employed to ensure stable signal transmission between the sensor and the oscilloscope. To further suppress external electromagnetic interference, conductive tape was applied to the FPC for shielding, while the PCB was designed with large‐area copper planes for grounding. The oscilloscope was connected to a computer via the VISA communication protocol, and signal data were acquired and recorded using a Python‐based program.

### Simulation and Visualization

The simulation and modeling were conducted using the ANSYS 2025 Student R1 to analyze the mechanical behavior of the sensing device under various loading conditions. Upon completion of the simulations, the raw data were exported and reprocessed using external plotting tools to ensure clarity and consistency in presentation. 3D modeling and visualization were performed using Blender 4.2. Data processing and graphical analysis were carried out with OriginLab Pro 2025 learning edition. Optical characterization of the structural features was conducted using an Olympus BX53M optical microscope. The SEM images were captured by ZEISS Gemini 500.

### Mechanical Test Setup

Mechanical loading was applied using a multi‐axis displacement stage system (KA200 and KA100Z, Zolix Instruments Co., Ltd., Beijing). These displacement stages offer high positioning precision, with closed‐loop resolution reaching up to 1 µm.

To evaluate the transient voltage response of the commercial PVDF film, a drop‐weight test was employed. Standard steel weights of different masses (5 , 10 , 20 , 30 , 50 , 80 , and 100 g) were released freely from a fixed height of ≈2 cm onto PVDF samples cut into 1 cm × 1 cm pieces, thereby generating instantaneous impacts. The weights were lifted by a gripper mounted on a vertical displacement stage and released under computer control via a Python script, ensuring consistent and repeatable impact conditions across trials.

For the device characterization, a series of contact probes was fabricated to induce piezoelectric responses. Generally, for sliding‐based characterization, the sensing device was mounted on a manual lifting platform. A 3D‐printed contact probe was used to slip over the sensing device. The whole test system is shown in Figure S17 (Supporting Information). The displacement stage motion was controlled via the upper‐computer software. Unless otherwise specified, all sliding tests in this study were conducted at a speed of 2 mm s^−1^. In normal force tests, the 3D‐printed contact probe was mounted on the tip of a force gauge (Mark‐10 Series 7), which was fixed onto the vertical stage. The applied normal force was monitored using real‐time readings from the gauge.

In vibration tests, a piezoelectric actuator (material type P2T5, resonance frequency 50 kHz) purchased from the e‐commerce platform Taobao (Jiaming Electronics) was used as the contact probe. The actuator was driven by preset voltage waveforms generated via a USB‐6349 data acquisition device (National Instruments), enabling excitation at varying frequencies.

Mechanical durability of the device was evaluated under both tangential and normal loading conditions. For tangential loading, the device was brought into contact with the contact probe and subjected to lateral sliding motions at a constant speed of 10 mm s^−1^, with the contact depth controlled to half of the pillar height. For normal loading, a compressive force of 3 N was applied perpendicularly to the device at a constant speed of 10 mm s^−1^. In both cases, cyclic tests were carried out for over 1200 cycles. To assess stability, output signals were recorded during the initial 0–200 cycles and again during cycles 1000–1200, allowing comparison of the device performance before and after prolonged operation.

For edge perception experiments, the contact probe consisted of a microfabricated silicon wafer featuring 1D surface textures with alternating ridges and grooves.

For normal force and vibration characterizations, the vertical height of the test block was adjusted stepwise using the displacement stage at its maximum resolution, ensuring precise contact with the sensing surface.

### The Fabrication of the Contact Probe

For the edge distribution characterization experiments, 1D periodic surface patterns were fabricated on silicon wafers using direct laser writing lithography (Heidelberg uPG501). The photoresist used for patterning was AZ2035. Following exposure and development, deep reactive ion etching (DRIE, Oxford Estrelas) was performed to produce structures with a depth of ≈ 100 µm.

For the other sliding‐based characterization, a 3D‐printed contact probe (Resin, Wenext, 8200Pro) was used to contact the sensing device and induce piezoelectric responses. To discuss the effect of multi‐material environments, additional contact probes were fabricated using a silicone molding process, based on the same geometry of the original resin probe. The materials included PMMA, wax, PDMS (mixing ratio A:B = 10:1), Dragon Skin 30 (A:B = 1:1), and Ecoflex 30 (A:B = 1:1). The elastic modulus of those materials are listed in Table S2 (Supporting Information). The silicone molding process ensured consistency in probe dimensions and surface details across different materials, thereby minimizing potential fabrication tolerances that could otherwise affect the experimental results. All experiments were conducted under room‐temperature conditions to ensure that the wax did not soften during testing.

### Scenarios Test Setup

In all application scenarios, the sensor was integrated into a custom‐designed robotic gripper. The gripper was mounted at the end effector of a UR3 robotic arm (Universal Robots). The robotic arm could communicate with a Python‐based program on the computer, enabling motion responses based on signals acquired from the oscilloscope. Through programmed motion control of the robotic arm, various active exploration modes were implemented to investigate the sensor's performance under dynamic interaction conditions.

To evaluate liquid discrimination, two types of exploratory actions were implemented: tapping and shaking. To enhance the separability of spectral features, repeated shaking motions were introduced. Each shaking cycle was defined by a programmed sequence of wrist joint rotations (−3.75°, +7.5°, −3.75°, back to −7.5°) at a speed = 40°/s. For consistency and safety, experiments were conducted with 3 and 6 shaking cycles, balancing signal enhancement with experimental efficiency.

The amplitude and speed of shaking were set to reliably excite liquid vibrations while avoiding spillage or accidental slippage of the cup from the gripper. These parameters were kept constant across trials, ensuring repeatability of the experiments. The container type and its placement were kept fixed throughout the experiment.

### Algorithms & Analysis Methods

For all experiments with the robot's dexterous hand, signal acquisition was synchronized with the onset of programmed robotic actions (sliding, grasping, or shaking). The duration of each acquisition window was determined by the corresponding exploratory behavior and terminated upon completion of the action. The recorded signal segments were subsequently processed offline, where the corresponding feature extraction method was applied to map the extracted features to object properties. The present work focuses on analyzing characteristic responses during exploratory behaviors, rather than executing real‐time tasks.

In the Robotic Cap‐Unscrewing Task, the recorded signals were characterized by time‐domain spikes generated during sliding across the cap. Multiple angular conditions were tested in small increments, and the peak amplitudes of spikes across segments were compared. Because the angular response of the device is highly nonlinear, the difference in peak amplitudes between adjacent angles becomes much larger near orthogonal orientations than at other regions. On this basis, the threshold was defined from the relative amplitude differences, providing a reliable criterion for orientation discrimination and ensuring better generalizability across similar scenarios.

For liquid classification, the recorded signals were analyzed using the STFT. From the time–frequency representation, multiple statistical features were extracted, including energy decay rate, mel‐spectrogram distribution, and chroma‐based harmonic content. These feature vectors were standardized and reduced to two dimensions using PCA or t‐SNE for visualization. To assign liquid types, clustering was performed using K‐means.

### Statistical Analysis

In this work, the data were expressed as the “mean ± standard deviation”. Error bars in all figures for average periods are the standard deviations obtained from five individual periods unless otherwise stated. The structural comparisons in Figure 3A were based on three samples for each category. All data were analyzed and performed by Origin Software and Python.

## Conflict of Interest

Part of the research is patent pending.

## Author Contributions

Y.Z.Z., J.Q.Z., and X.M.L. conceived the research concept. Y.Z.Z., J.Q.Z., and L.Y.L. conducted the fabrication and characterization of the sensing device. Y.Z.Z. and J.Q.Z. contributed to all data analysis. Y.Z.Z., M.X.H., Z.Y.Z., and X.M.L. contributed to robots’ dexterous hand integration and related examinations. Y.Z.Z., X.M.L., and Z.L.L. contributed to the script for data acquisition and processing. X.C.G. provided equipment support for direct laser writing lithography and DRIE. X.M.L. supervised the project and acquired funding. All authors have read and approved the final manuscript.

## Supporting information



Supporting Information

Supplemental Video 1

Supplemental Video 2

Supplemental Video 3

## Data Availability

The data that support the findings of this study are available from the corresponding author upon reasonable request.
