# Supplementary material for: Artificial Tactile Perception System for Exploring Internal and External Features of Objects via Time‐Frequency Features
Source: Adv Sci (Weinh). 2025 Nov 11;13(14):e09928. doi: 10.1002/advs.202509928 (PMC12970215; doi:10.1002/advs.202509928)
Supplement: Supplementary file 1 — Supporting Information [file ADVS-13-e09928-s003.docx]

Supporting Information

Artificial tactile perception system for exploring internal and external features of objects *via* time-frequency features

Yuanzhi Zhou^1^, Jingqi Zhang^1^, Linyi Li^1^, Muxing Huang^1^, Ziyi Zhang^1^, Xingmin Lu^1^, Zilan Li^1^,Xuchun Gui^2^, Xinming Li^1,^*

**Table S1-S2**

**Figure S1-S14**

**Video S1-S3**

**Table S1. Quantitative comparison of devices**

| **Ref.** | **Sensor Type** | **Force Sensitivity** | **Spatial Resolution** | **Bandwidth** | **Spectral Resolution** |
| --- | --- | --- | --- | --- | --- |
| Ours | Piezoelectric | 0.02 N | ~100 µm | 0-2000 Hz | 1 Hz |
| [1] | Magnetic–Hall effect | < 1 N | 0.1 mm | – | – |
| [2] | Magnetic–Hall effect | ~1 mN | Fingertip-level resolution | ~10 to 10^2^ Hz | – |
| [3] | Magnetic–Hall effect | 7.56 kPa^-1^ | Angular spatial resolution | – | – |
| [4] | Camera + microphone + pressure MEMS | Normal: 1.01 mN; Shear:1.27 mN | 7 μm | up to 10 kHz | – |
| [5] | Capacitive | < 1.5 N | 3.8 mm (inferred by array pitch) | up to 300 Hz | – |
| [6] | Triboelectric + resistive | < 1 N | 500 μm (inferred by electrode diameter) | – | – |

**Table S2 Materials Tested in Edge Detection Experiment**

| **Material** | **Elastic Modulus** |
| --- | --- |
| Resin | 2600 MPa[7] |
| PMMA | 1800-3100 MPa[8] |
| Wax | ~314 ± 35 MPa[9] |
| PDMS | ∼1.25 to ∼2 MPa[10] |
| Dragon Skin 30 | 0.74 MPa[11] |
| Ecoflex 30 | ∼20 to ∼35 kPa[12] |


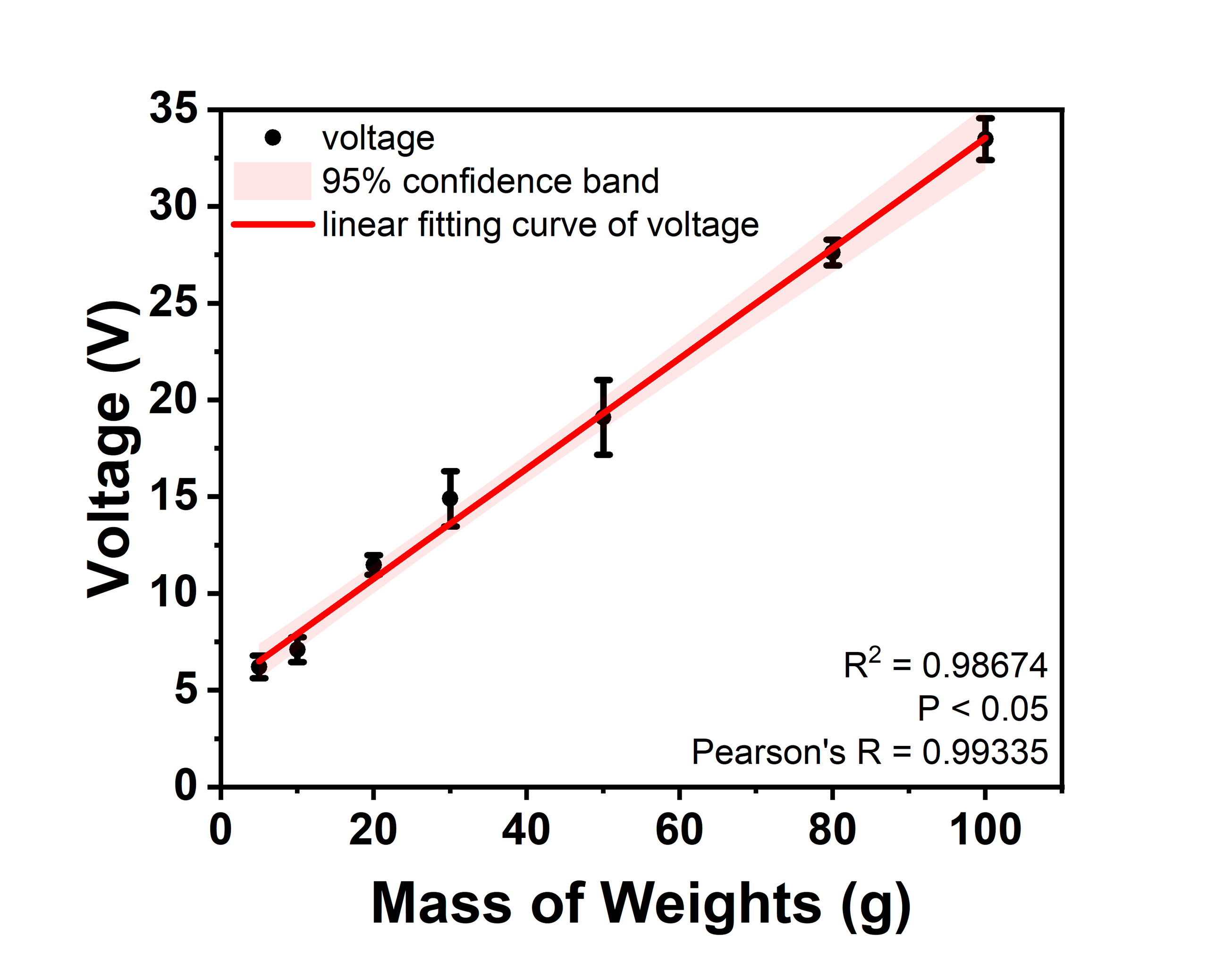


**Figure S 1** The intrinsic piezoelectric response of the PVDF layer


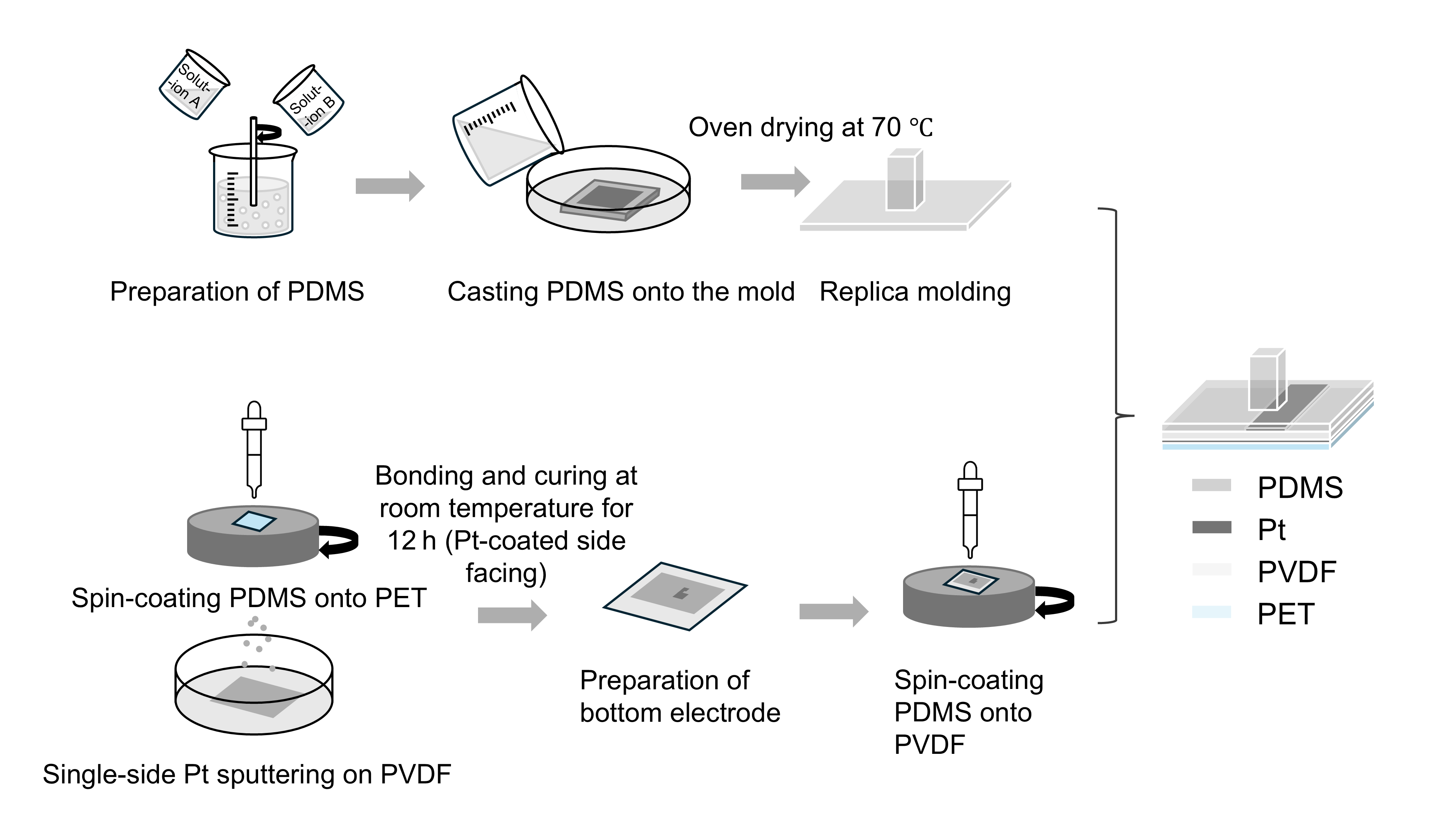


**Figure S 2** The fabrication process of the sensing device

**
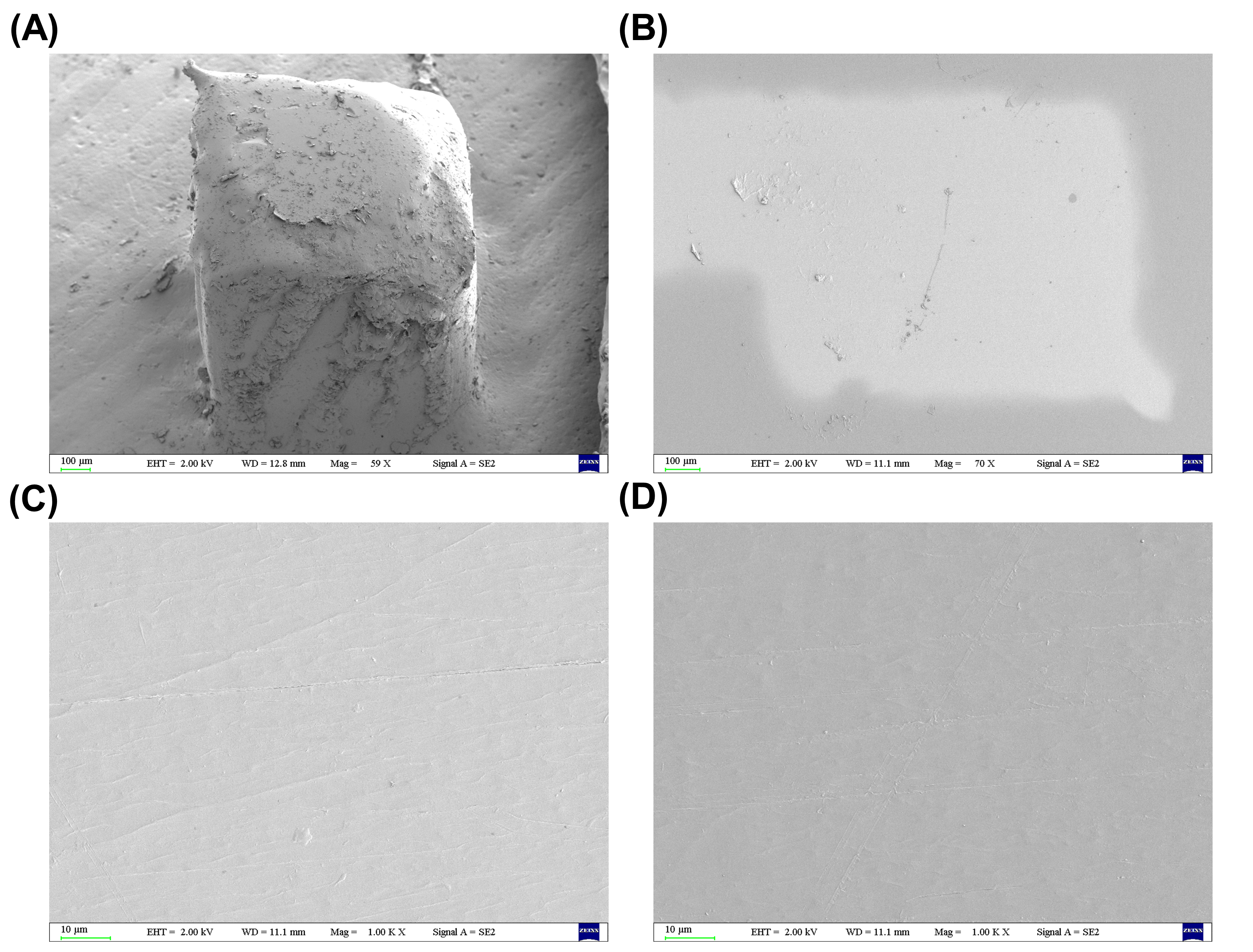
**

**Figure S 3** The SEM image of (A) PDMS pillar (w:h = 1:1 mm); (B) the Pt electrode; (C) the surface of the Pt electrode; (D) the surface of the PVDF film.


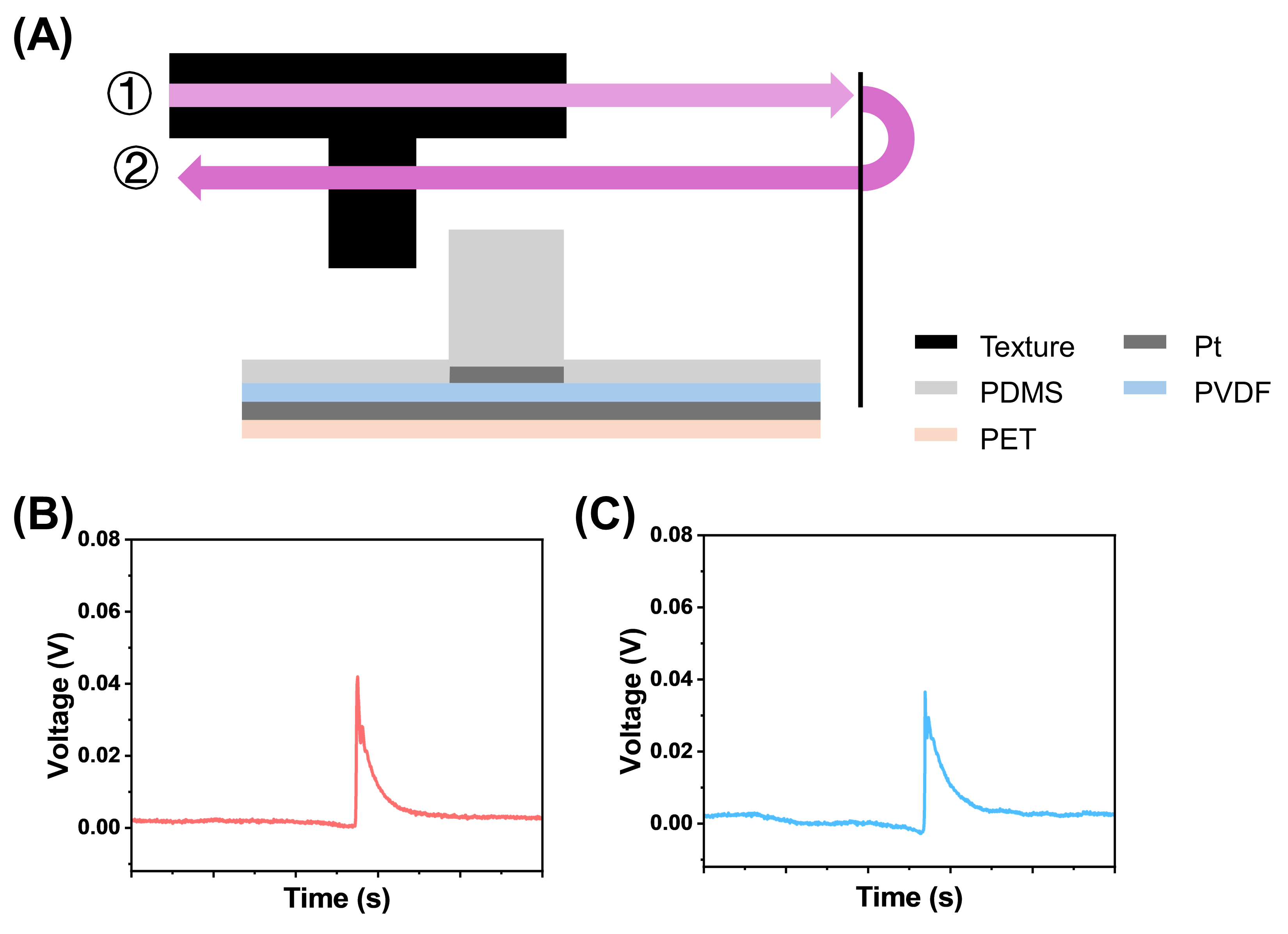


**Figure S 4** (A) Test Schematic Diagram; (B)The signals from the centered-located-design of the top electrode and pillars.


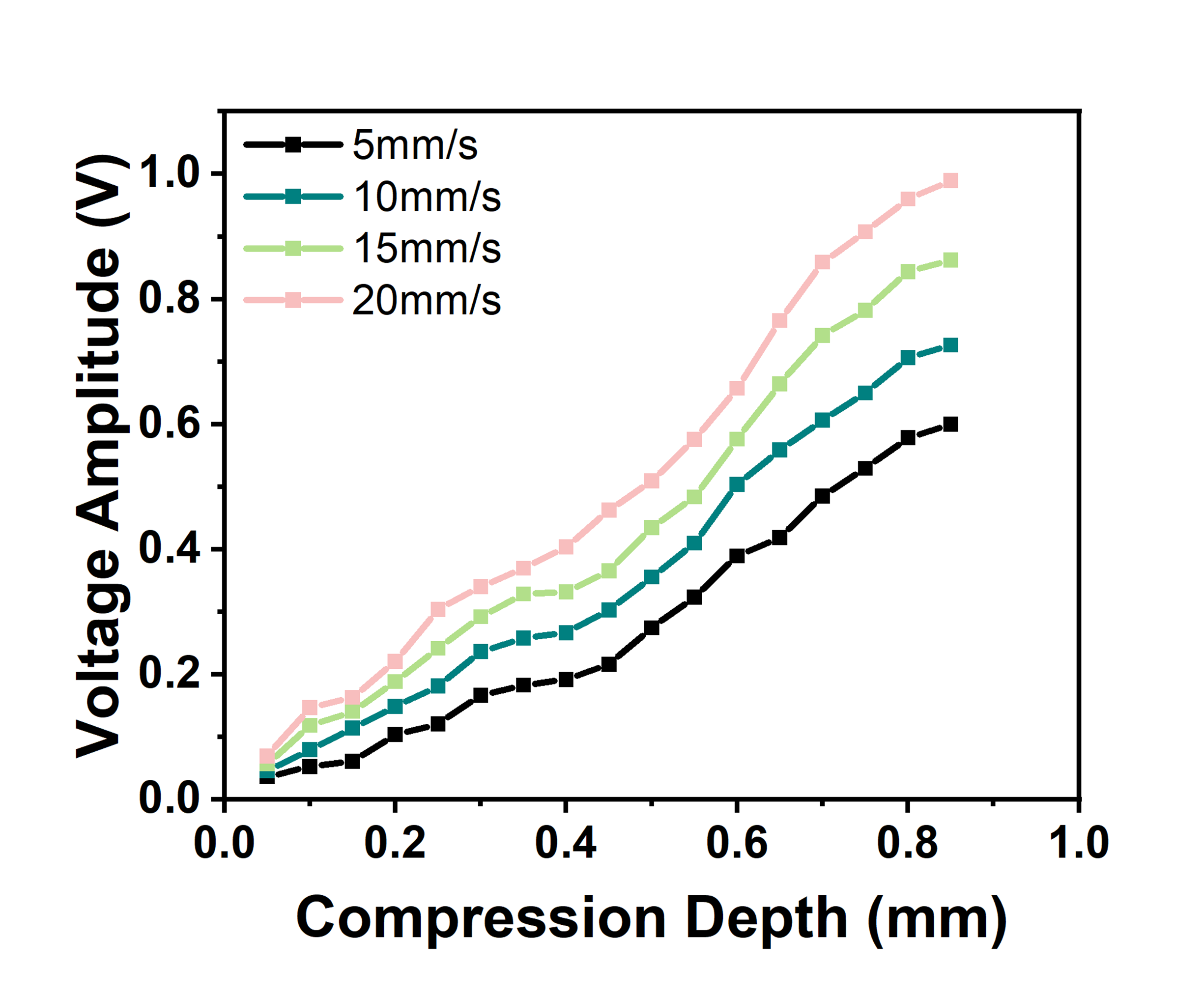


**Figure S 5** Response to different sliding speeds and contact depth


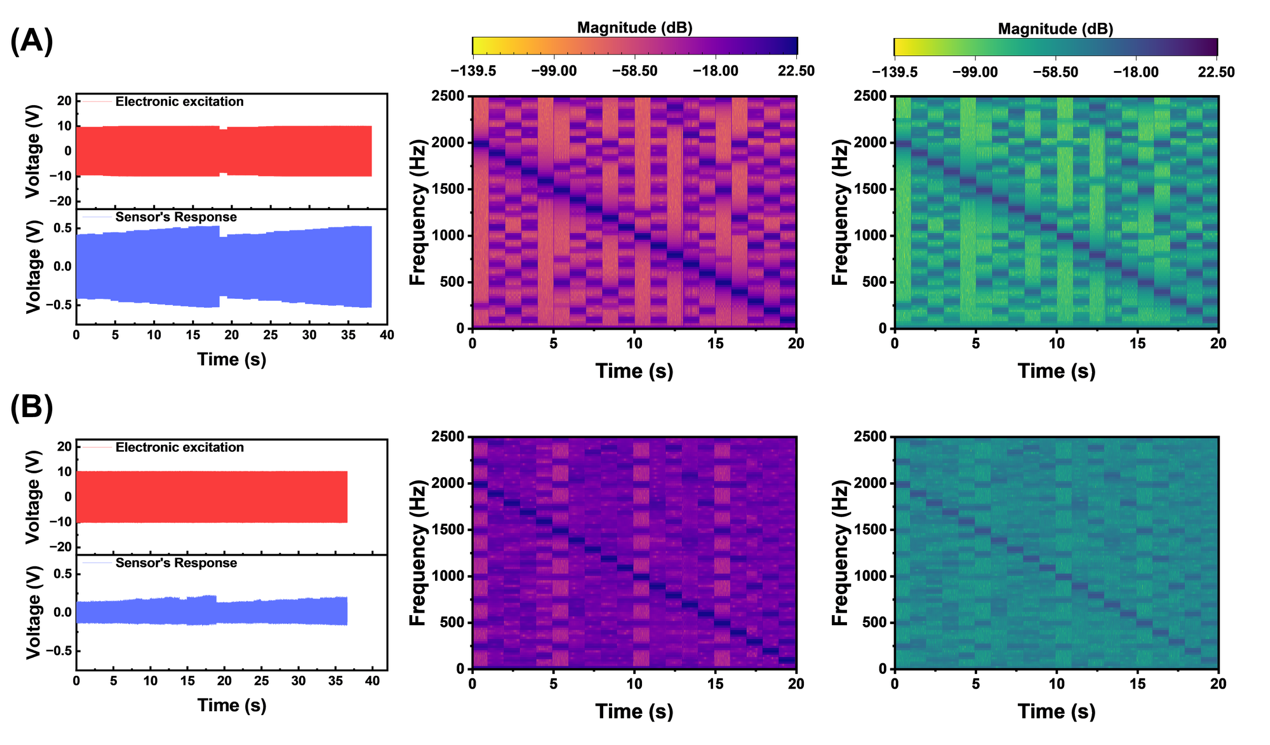


**Figure S 6** (A) Response to vibration from 100 Hz to 200 Hz from w:h = 1:1 (mm) in the deviation-located-design of the top electrode and pillars; (B) Response to vibration from 100 Hz to 200 Hz from w:h = 1:1 (mm) in the centered-located-design of the top electrode and pillars.


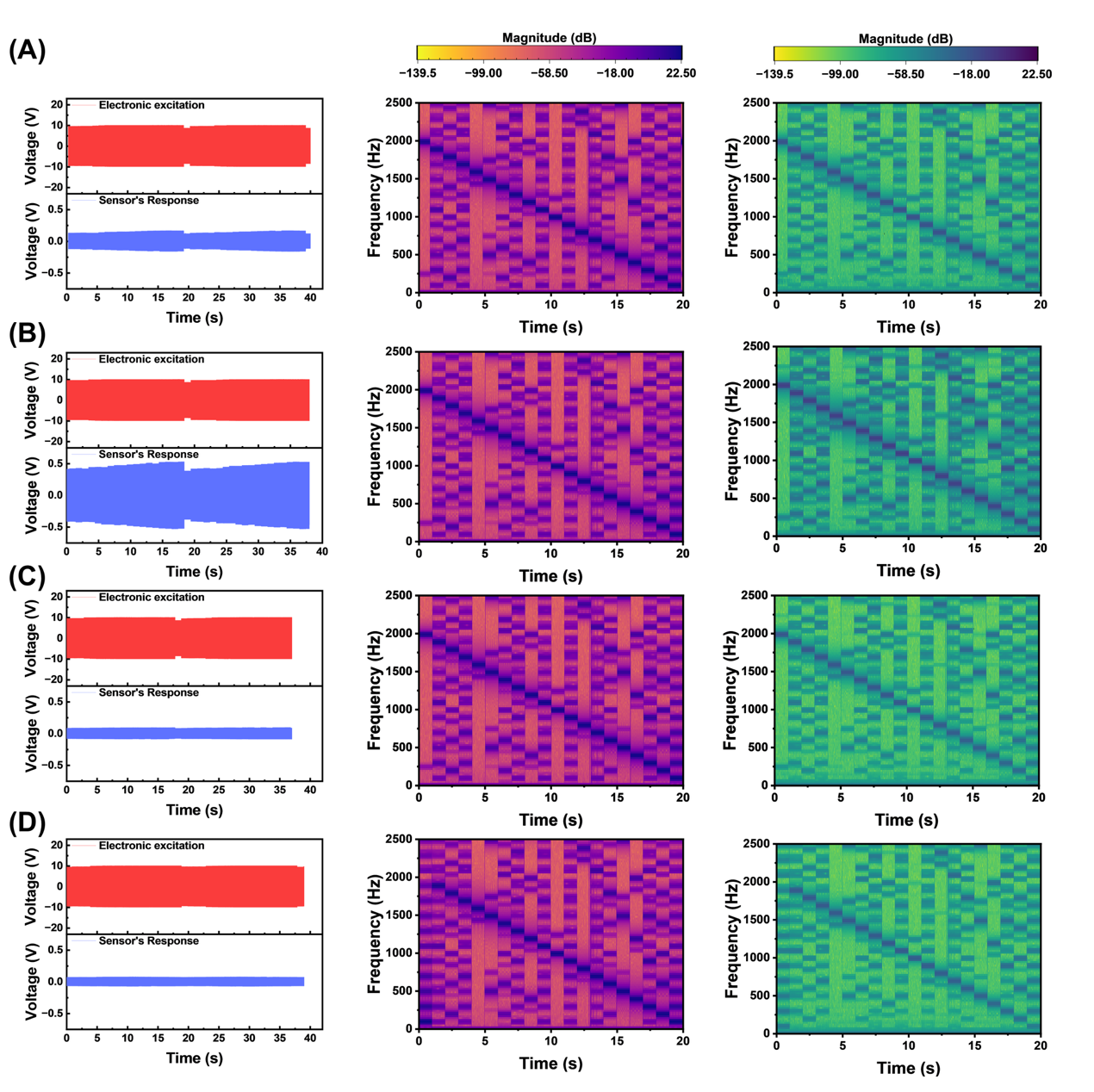


**Figure S 7** (A) Response to vibration from 100 Hz to 200 Hz from w:h = 1:0.5 (mm) in the deviation-located-design of the top electrode and pillars; (B) Response to vibration from 100 Hz to 200 Hz from w:h = 1:1 (mm) in the deviation-located-design of the top electrode and pillars; (C) Response to vibration from 100 Hz to 200 Hz from w:h = 1:1.5 (mm) in the deviation-located-design of the top electrode and pillars; (D) Response to vibration from 100 Hz to 200 Hz from w:h = 1:2 (mm) in the deviation-located-design of the top electrode and pillars;


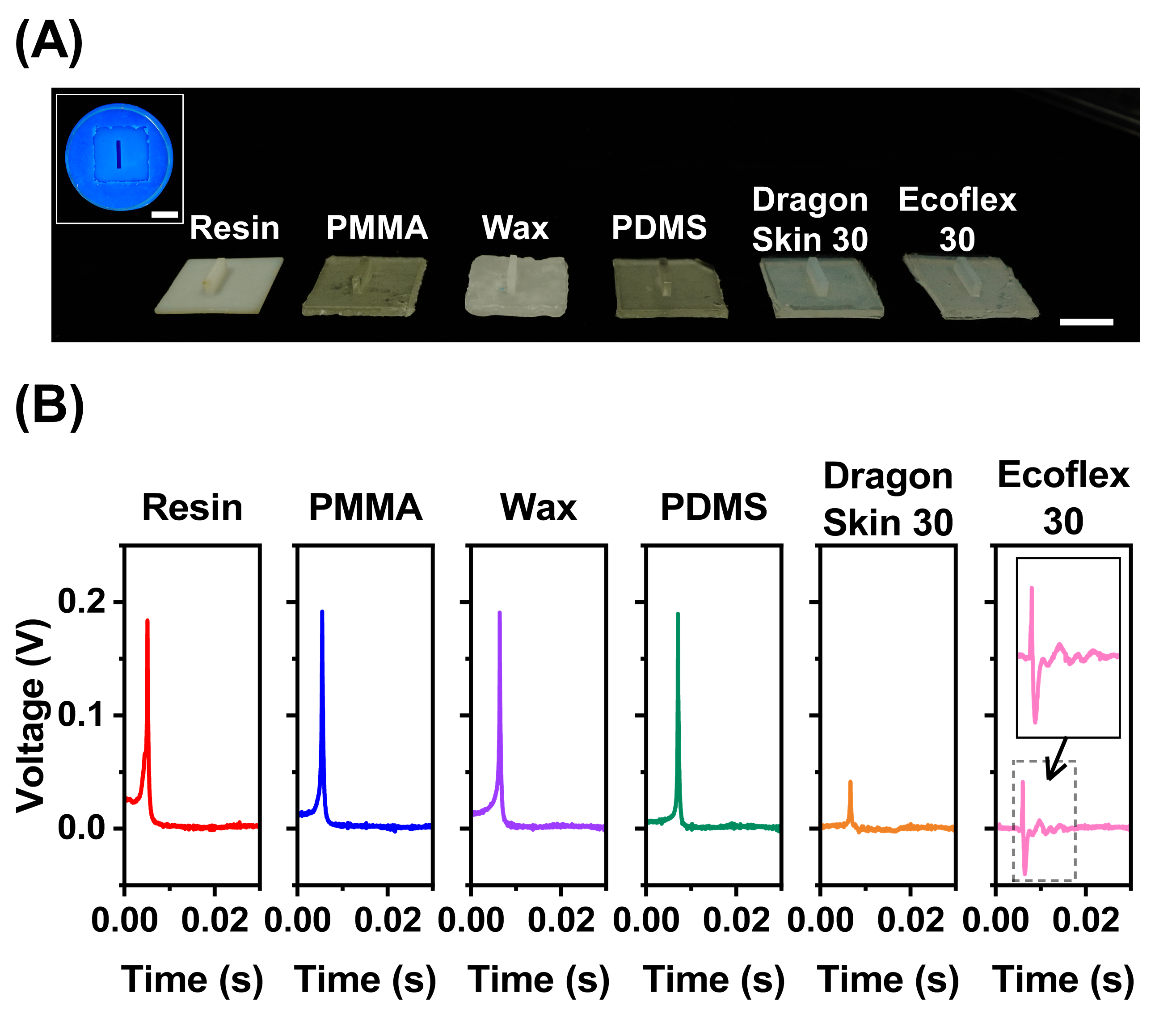


**Figure S 8** (A) The optical image of 6 kinds of contact probes (inset: the mold, scalebar = 1 mm); (B) The corresponding spikes in state 4.


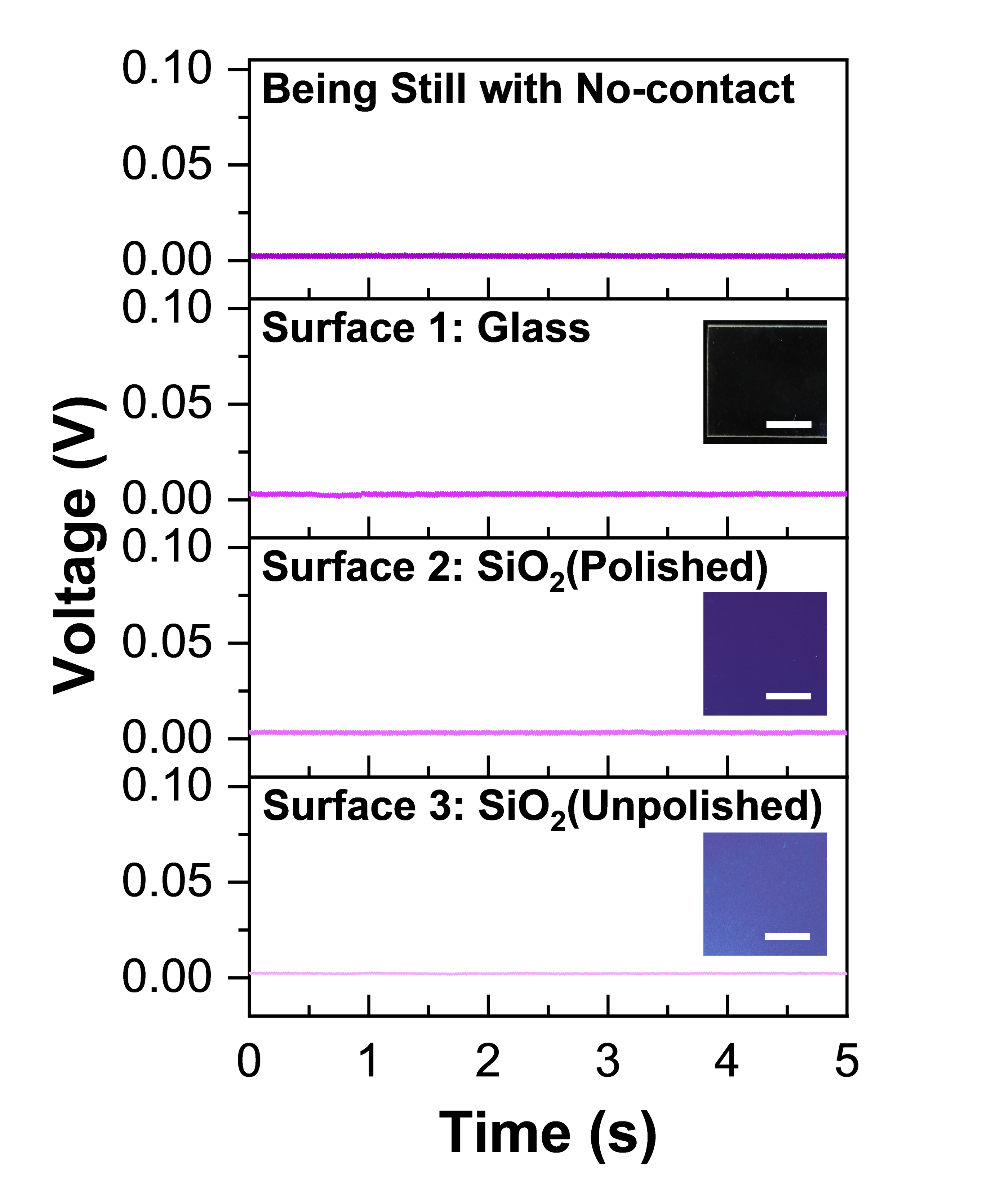


**Figure S 9** The responses to sliding on flat substrates, including glass slides, polished silicon surfaces, and unpolished silicon surfaces. Scalebar = 1 cm.


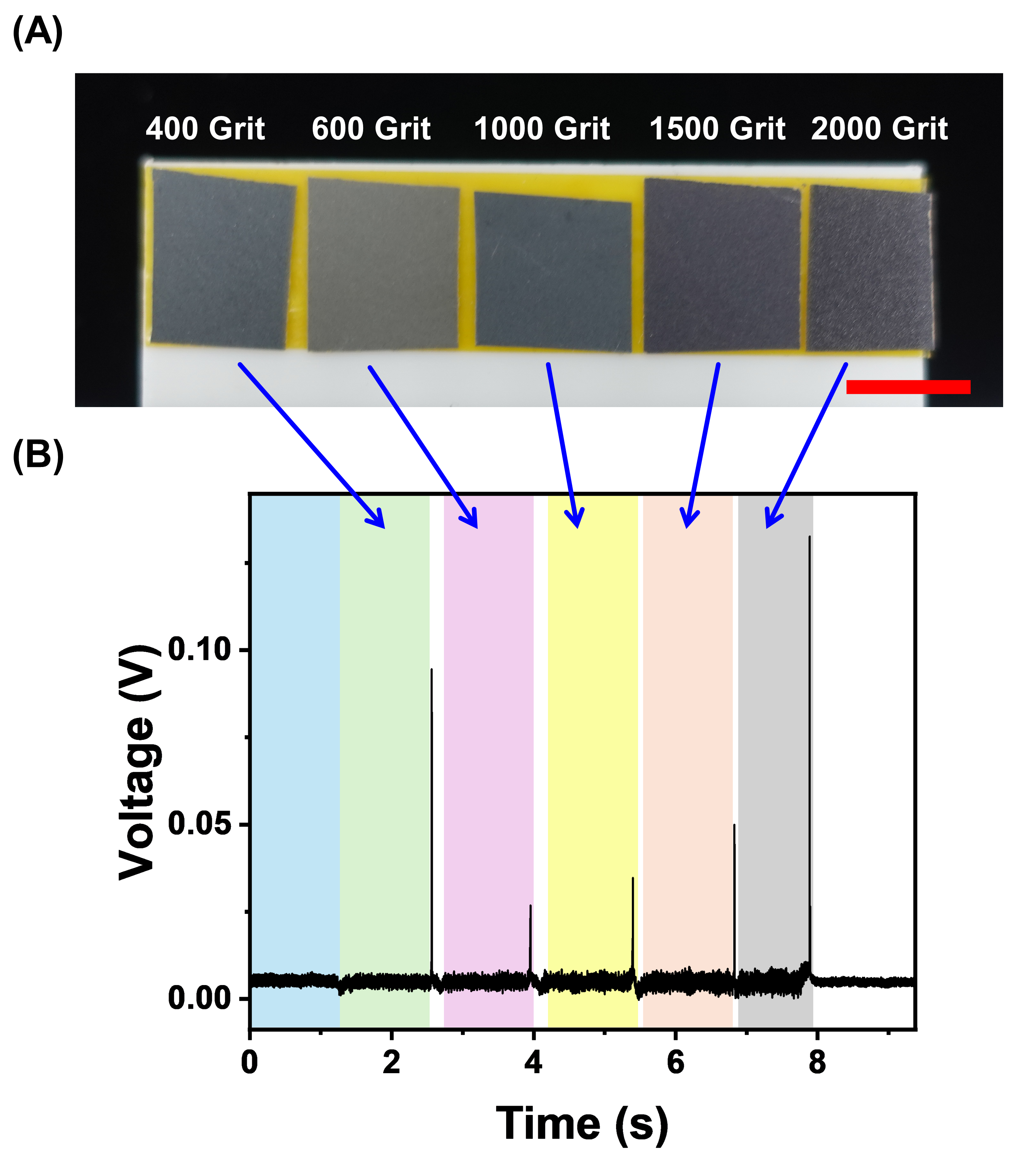


**Figure S 10** (A) The optical image of sandpapers of graded roughness ranging from 400 to 2000 grit; (B) The corresponding signal.


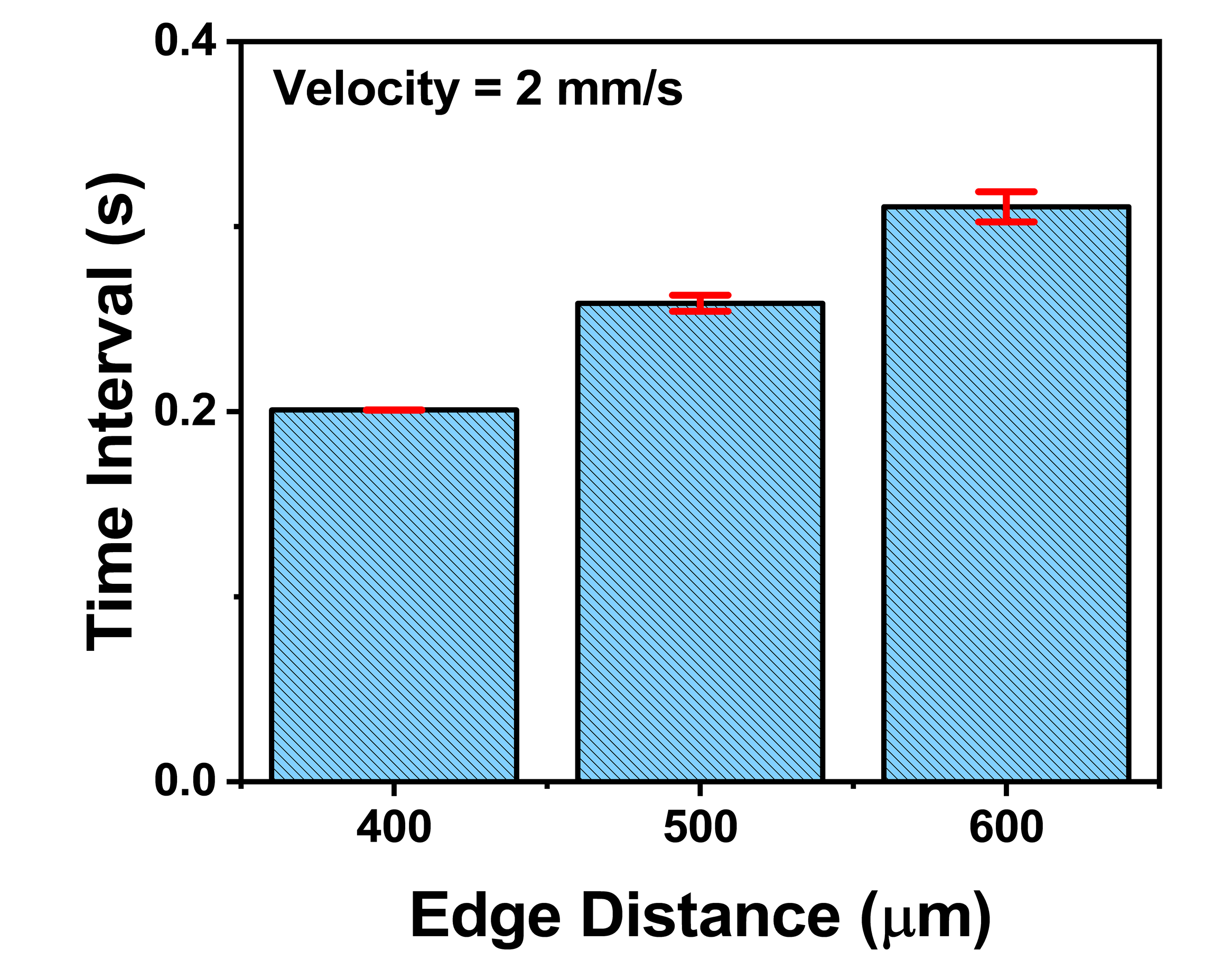


**Figure S 11** Responses to sliding on the surface structures with protrusion spacings of 400, 500, and 600 μm


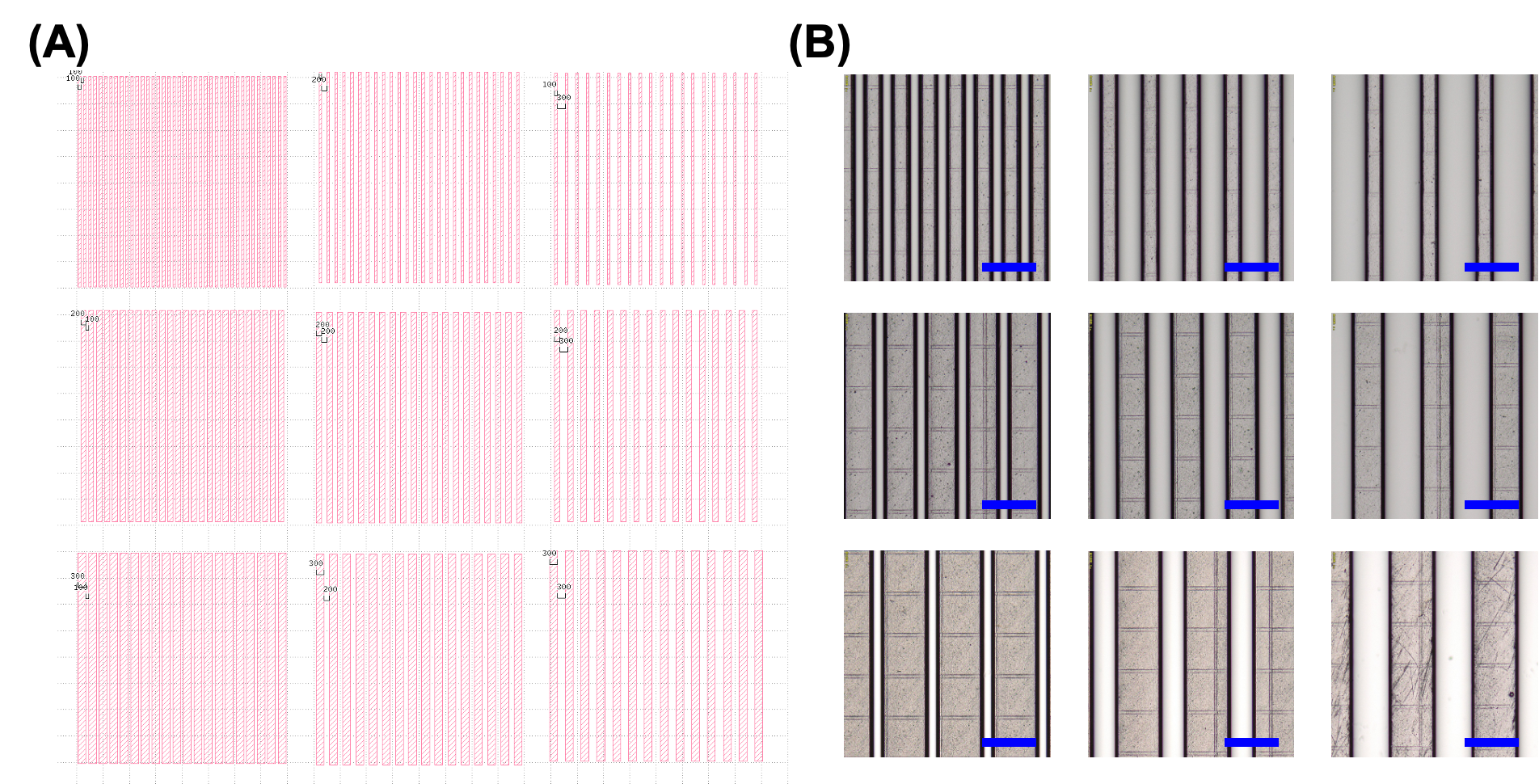


**Figure S 12** (A) Photolithographic layouts of different edge distribution patterns, where the shaded regions represent raised regions; (B) Corresponding optical microscope images of the fabricated microstructures are shown on the right. Scale bar: 400 µm.


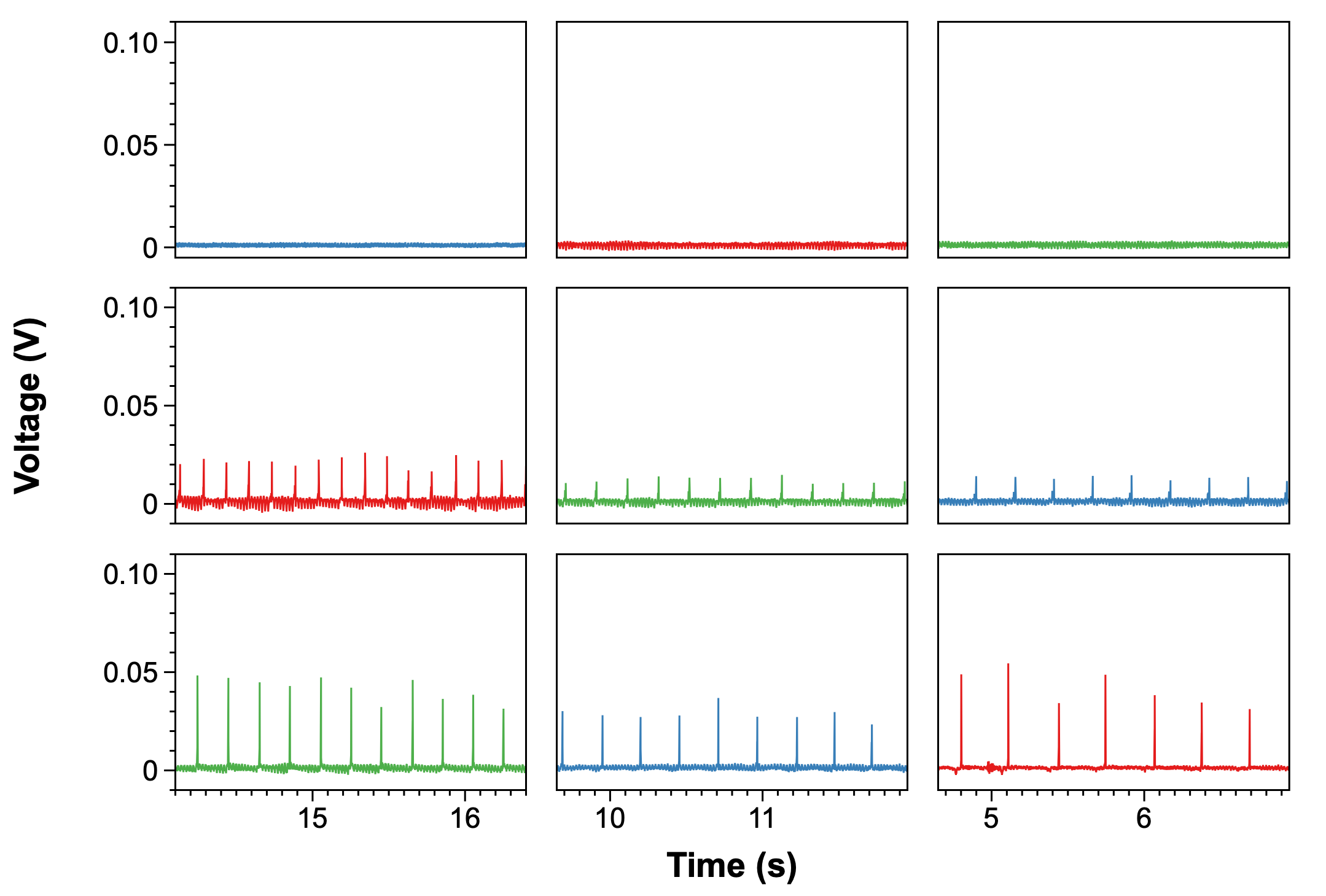


**Figure S 13** Corresponding Response to different edge distributions


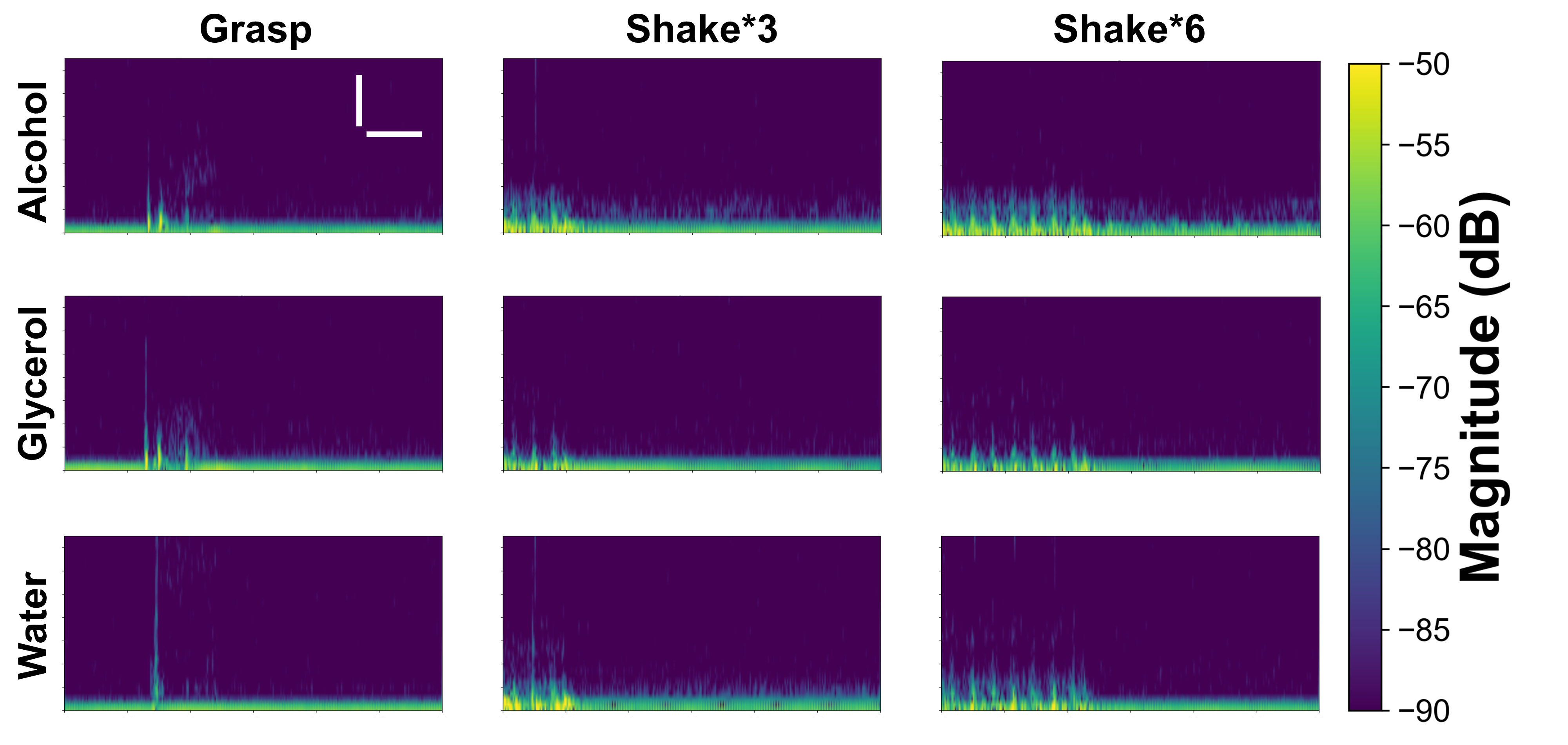


**Figure S 14** The frequency-domain responses under different stimulation behaviors. Vertical scale bar= 500 Hz, horizonal scale bar= 0.5 s


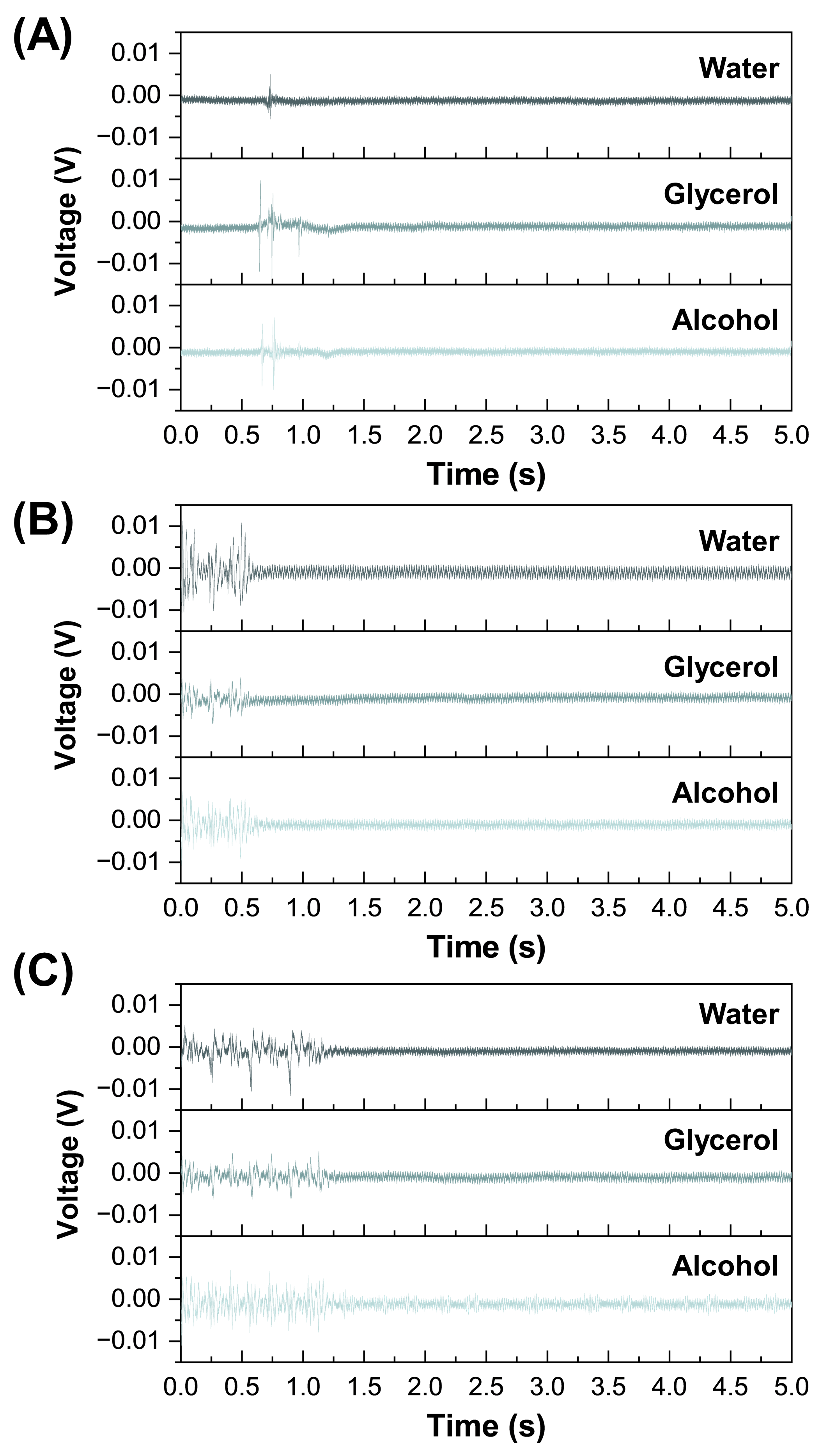


**Figure S 15** (A) The time-domain responses under grasp behaviors; (B) The time-domain responses under shaking*3 behaviors; (C) The time-domain responses under shaking*6 behaviors;


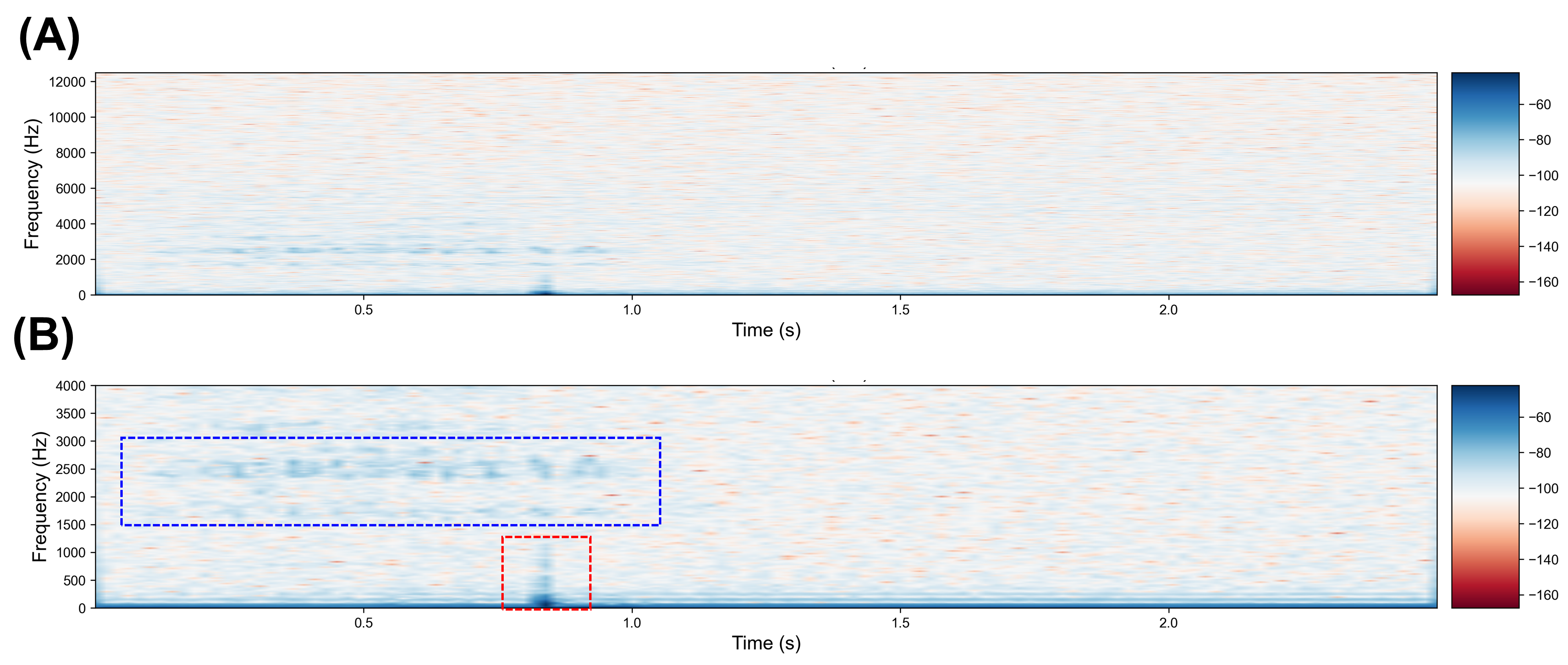


**Figure S 16** (A) The signals in the time-domain and STFT spectrum in the frequency range from 0-12500 Hz; (B) The signals in the time-domain and STFT spectrum in the frequency range from 0-4000 Hz. The blue dashed box highlights the spectral components associated with robotic arm motion noise, while the red box indicates the components induced by liquid oscillation.


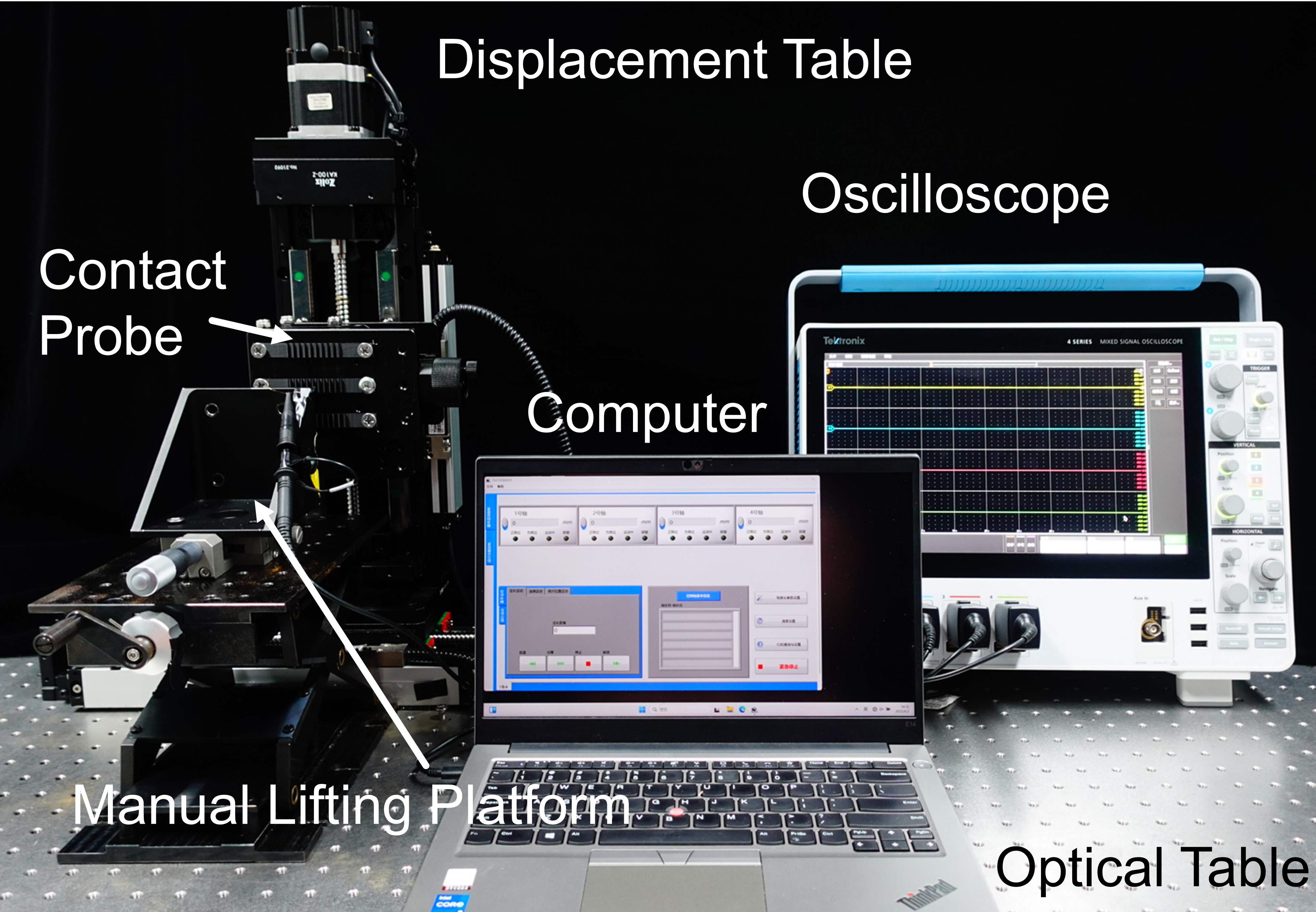


**Figure S 17** Mechanical test environment

**References**

[1] Yan, Y., Hu, Z., Yang, Z., et al., “Soft magnetic skin for super-resolution tactile sensing with force self-decoupling,” *Science Robotics* 6(51), (2021): eabc8801. https://doi.org/10.1126/scirobotics.abc8801

[2] Fang, D., Ding, S., Liu, Y., et al., “Revisiting the ‘Stick‐Slip’ Process via Magnetism‐Coupled Flexible Sensors with Bioinspired Ridge Architecture,” *Advanced Materials* 37(19), (2025) : 2417867. https://doi.org/10.1002/adma.202417867

[3] Ren, H., Yang, L., Chang, H., et al., “A robust and omnidirectional-sensitive electronic antenna for tactile-induced perception,” *Nature Communications* 16, (2025): 3135. https://doi.org/10.1038/s41467-025-58403-3

[4] Lambeta, M., Wu, T., Sengul, A., et al., Digitizing Touch with an Artificial Multimodal Fingertip, 2024. https://doi.org/10.48550/arXiv.2411.02479

[5] Huh, T. M., Choi, H., Willcox, S., Moon, S., Cutkosky, M. R., “Dynamically Reconfigurable Tactile Sensor for Robotic Manipulation,” *IEEE Robotics and Automation Letters* 5(2), (2020): 2562–2569. https://doi.org/10.1109/LRA.2020.2972881

[6] He, S., Dai, J., Wan, D., et al., “Biomimetic bimodal haptic perception using triboelectric effect,” *Science Advances* 10(27), (2024): eado6793. https://doi.org/doi:10.1126/sciadv.ado6793

[7] https://www.wenext.cn/static/material/52 (accessed September 10, 2025).

[8] https://www.mit.edu/~6.777/matprops/pmma.htm (accessed September 10, 2025).

[9] Craig, R. G., Eick, J. D., Peyton, F. A., “Strength Properties of Waxes at Various Temperatures and Their Practical Application,” *Journal of Dental Research* 46(1), (1967): 300–305. https://doi.org/10.1177/00220345670460013101

[10] Rubino, E., Ioppolo, T., “Young’s modulus and loss tangent measurement of polydimethylsiloxane using an optical lever,” *Journal of Polymer Science Part B: Polymer Physics* 54(7), (2016): 747–751. https://doi.org/10.1002/polb.23972

[11] Lo Presti, D., Dall’Orso, S., Muceli, S., et al., “An fMRI Compatible Smart Device for Measuring Palmar Grasping Actions in Newborns,” *Sensors* 20(21), (2020): 6040. https://doi.org/10.3390/s20216040

[12] Janardhana, R., Guler, Z., Akram, F., Jackson, N., Comparison of Mechanical Behavior of Macro and Micro-Sized Ecoflex 0030 and 0031, In *IMECE2024*, vol.10(2024): V010T13A001
